# Supplementary material for: Targeting Cellular Senescence to Enhance Human Endometrial Stromal Cell Decidualization and Inhibit Their Migration
Source: Biomolecules. 2025 Jun 16;15(6):873. doi: 10.3390/biom15060873 (PMC12191167; doi:10.3390/biom15060873)

Supplemental data S1: Full western blots for signaling data shown in Figure 5 (PPT file with all blot images)

Uncropped Western Blots Figure 5

Participant #1 12/18/2024 cell preparation date

Participant #2 12/18/2024

Participant #3 12/30/2024

Participant #4 12/2/2024

Participant #5 12/2/2024

Participant #6 12/10/2024

Participant #7 12/10/2024

Participant #8 11/11/2024

Image size
Individual blots: 3.2 X 4
Merged: 3.75X7 & 3.75X5

Figure 5. The differential effects of various flavonoid and non-flavonoid senotherapeutics on cell signaling pathways.

Supplemental data S 1

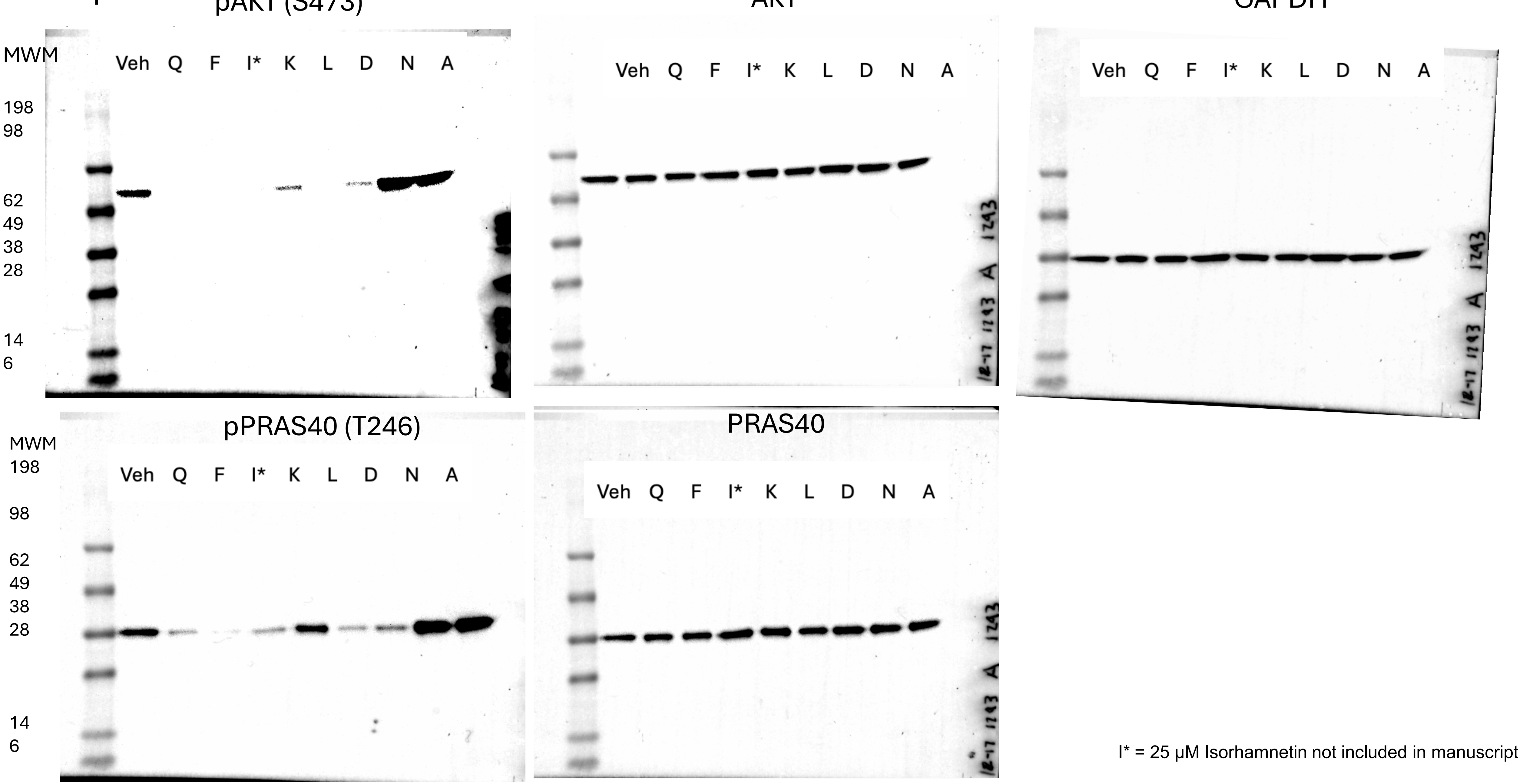

I* = 25 µM Isorhamnetin not included in manuscript

Figure 5. The differential effects of various flavonoid and non-flavonoid senotherapeutics on cell signaling pathways.

Supplemental data S 1

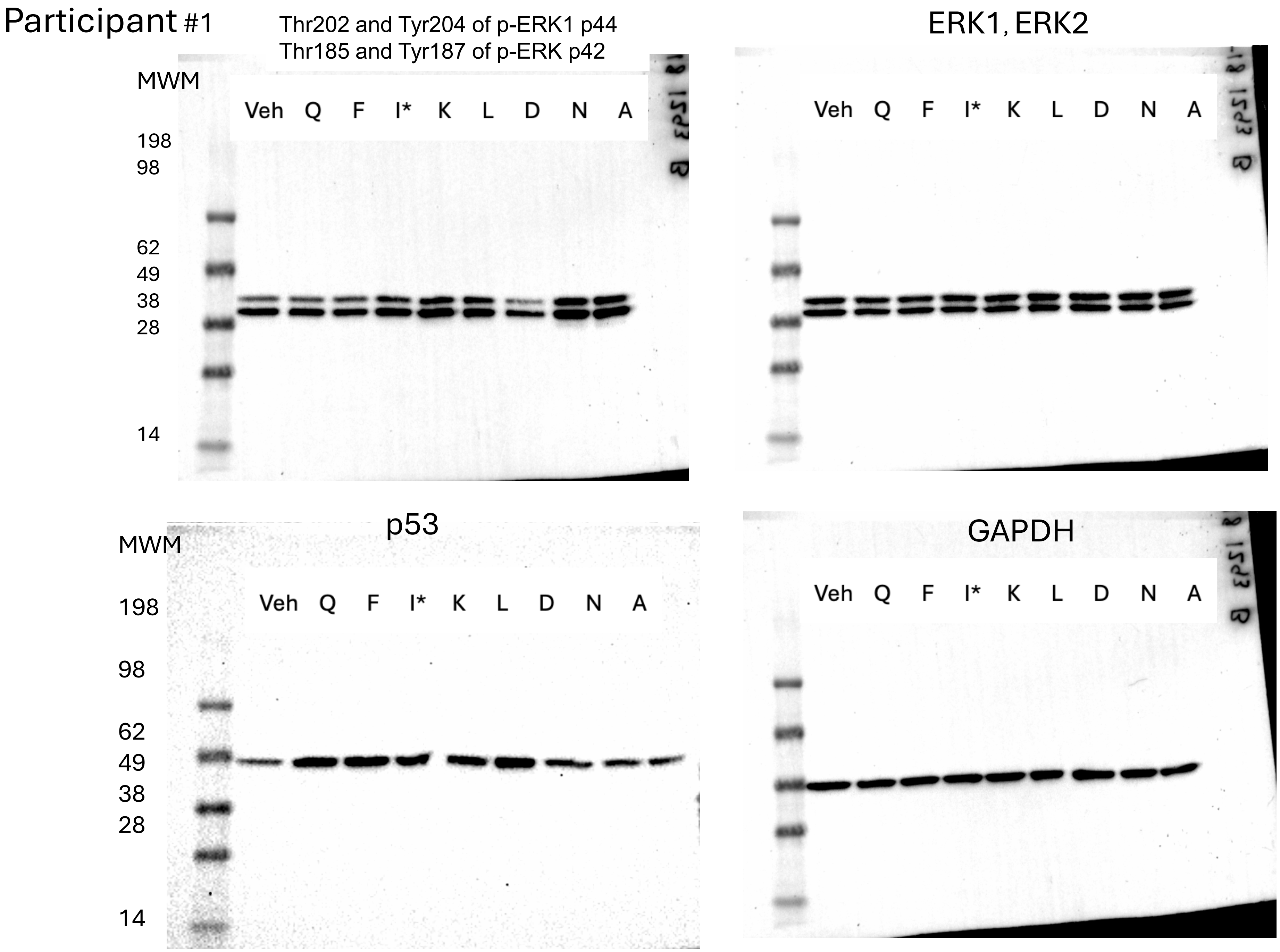

I* = 25 µM Isorhamnetin not included in manuscript

Supplemental data S 1

Figure 5. The differential effects of various flavonoid and non-flavonoid senotherapeutics on cell signaling pathways.

Participant #1

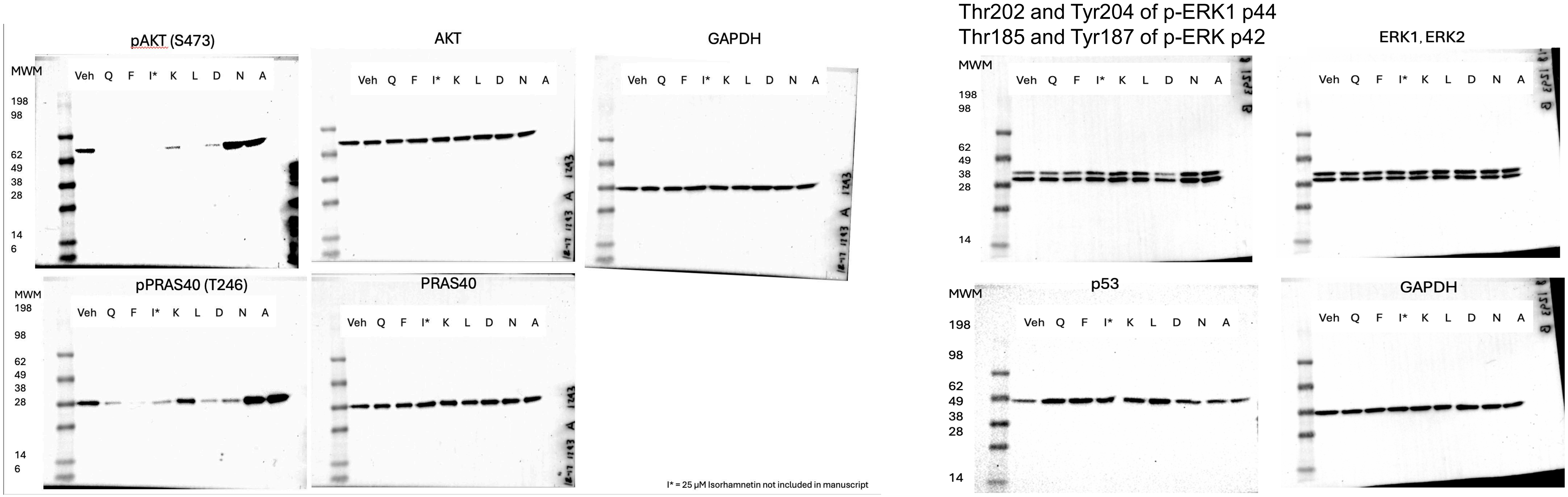

I* = 25 μM Isorhamnetin not included in manuscript

Figure 5. The differential effects of various flavonoid and non-flavonoid senotherapeutics on cell signaling pathways.

Supplemental data S 1

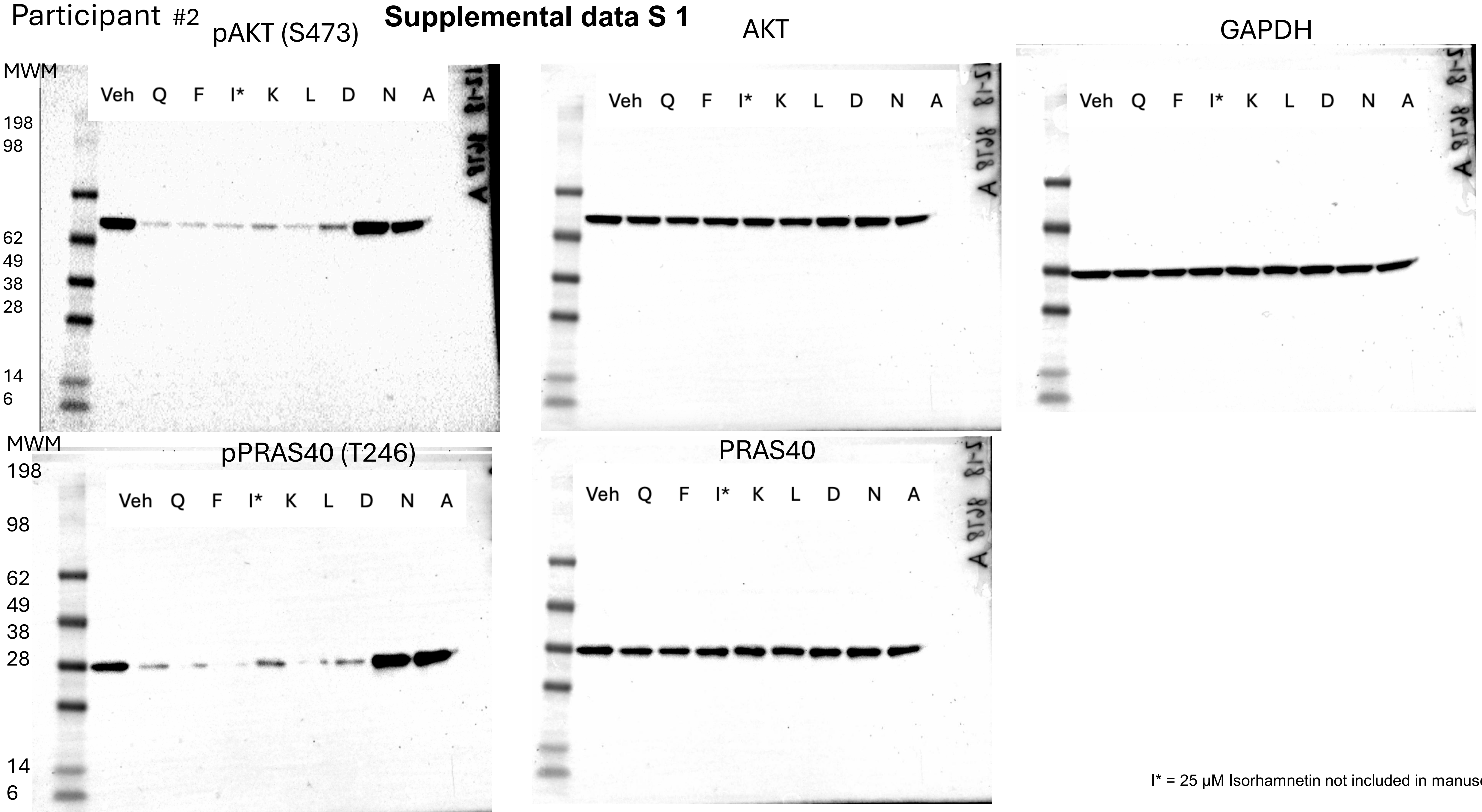

I* = 25 µM Isorhamnetin not included in manuscript

Figure 5. The differential effects of various flavonoid and non-flavonoid senotherapeutics on cell signaling pathways.

Supplemental data S 1

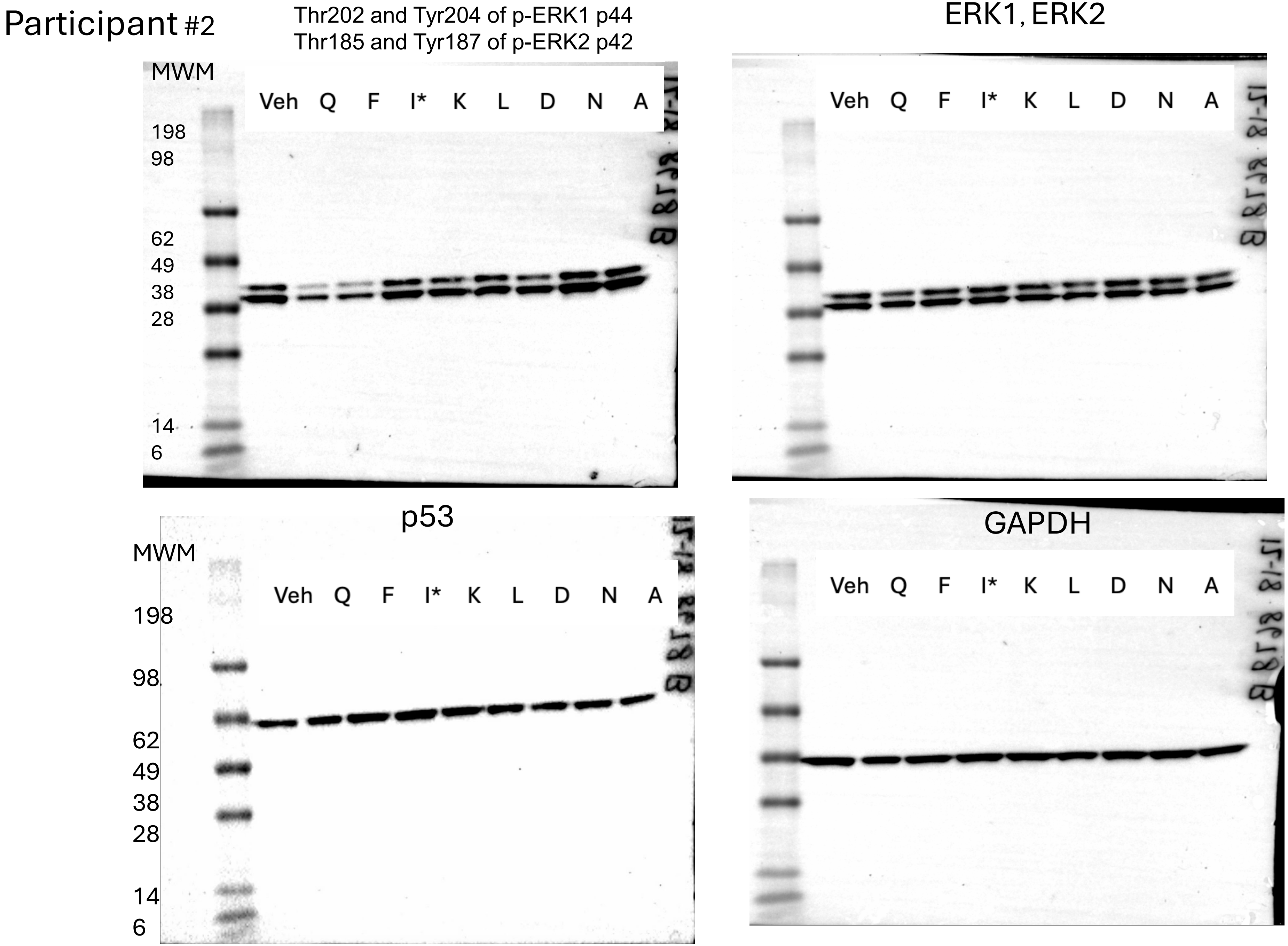

I* = 25 μM Isorhamnetin not included in manuscript

Supplemental data S 1

Figure 5. The differential effects of various flavonoid and non-flavonoid senotherapeutics on cell signaling pathways.

Participant #2

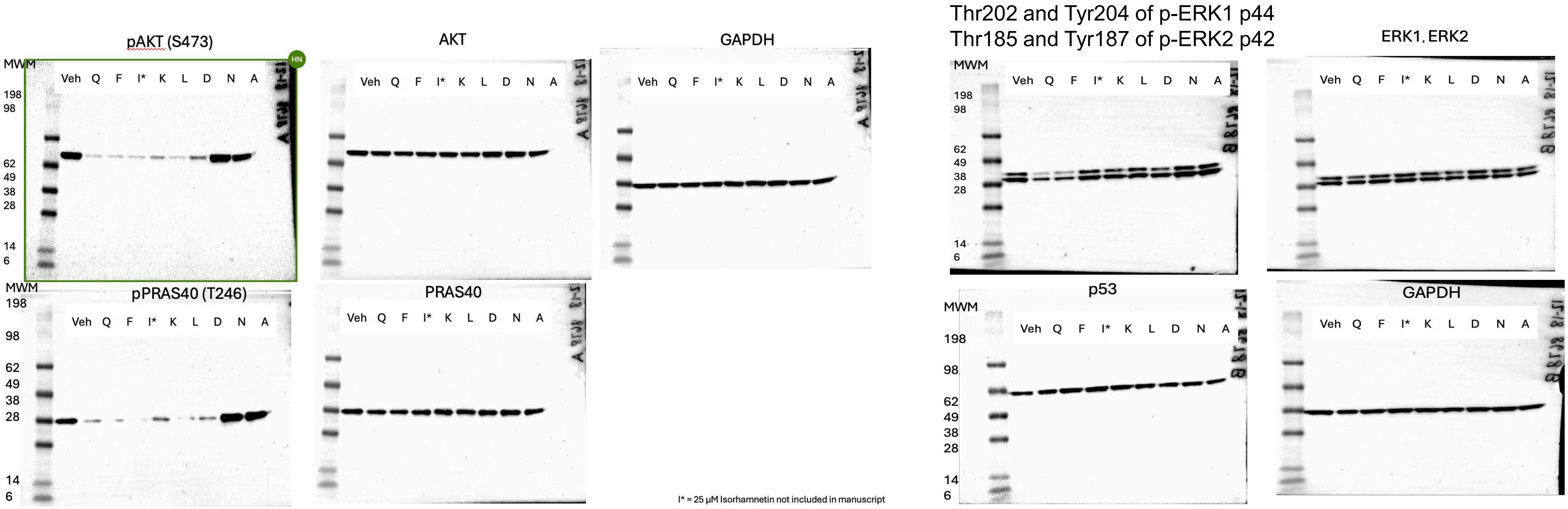

I* = 25 µM Isorhamnetin not included in manuscript

Figure 5. The differential effects of various flavonoid and non-flavonoid senotherapeutics on cell signaling pathways.

Participant #3

Supplemental data S 1

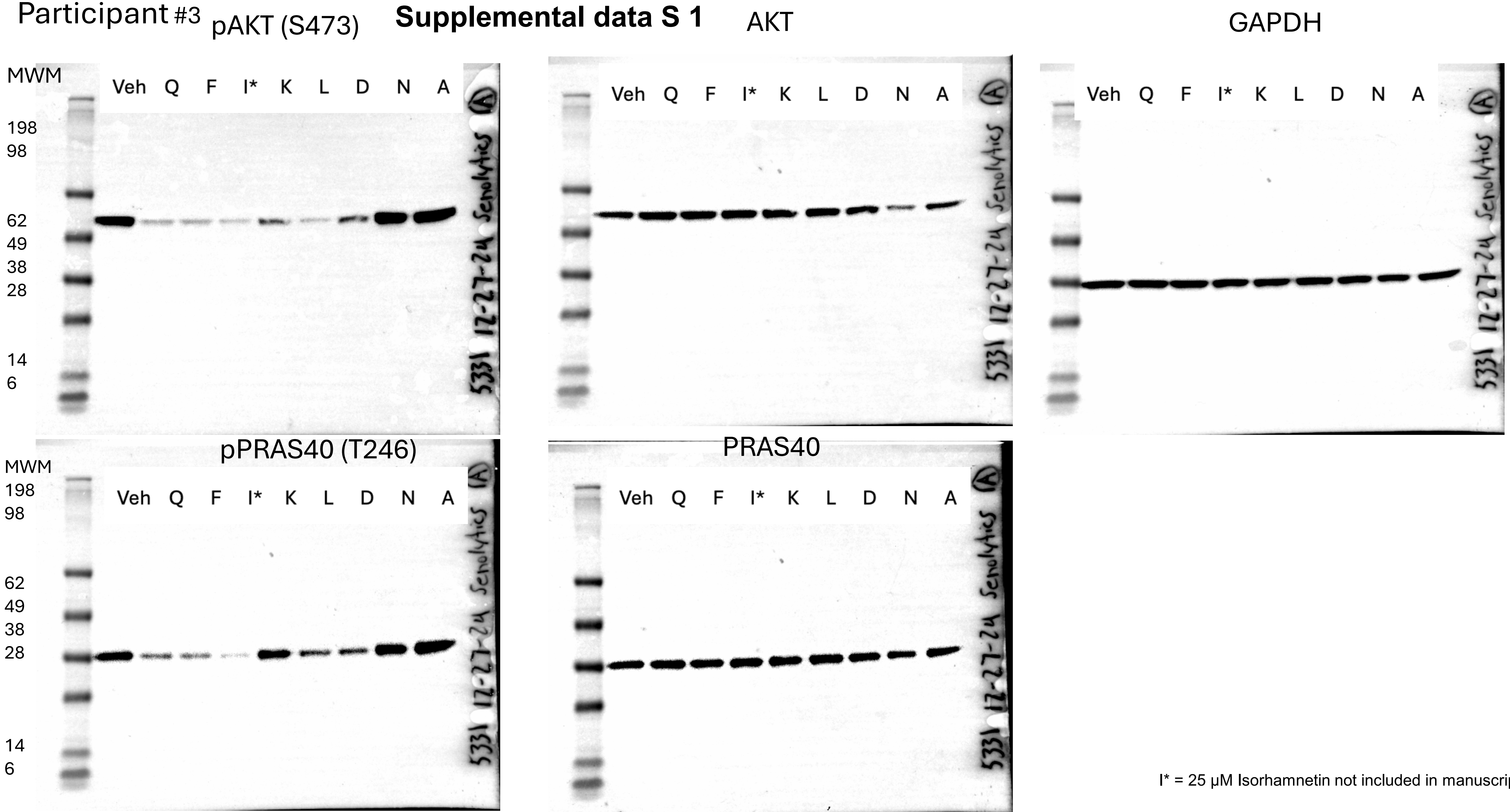

I* = 25 µM Isorhamnetin not included in manuscript

Figure 5. The differential effects of various flavonoid and non-flavonoid senotherapeutics on cell signaling pathways.

Supplemental data S 1

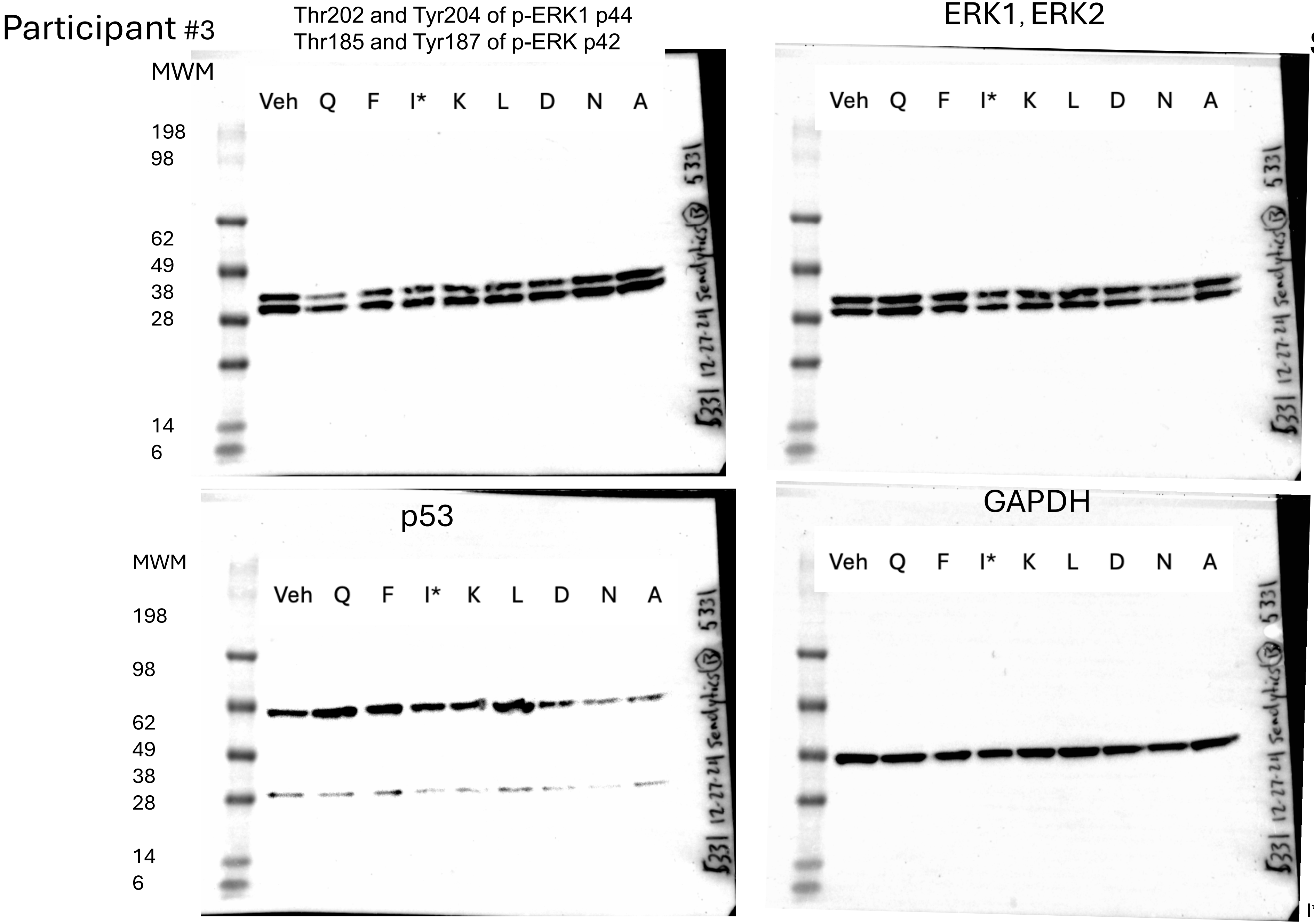

I* = 25 µM Isorhamnetin not included in manuscript

Supplemental data S 1

Figure 5. The differential effects of various flavonoid and non-flavonoid senotherapeutics on cell signaling pathways.

Participant #3

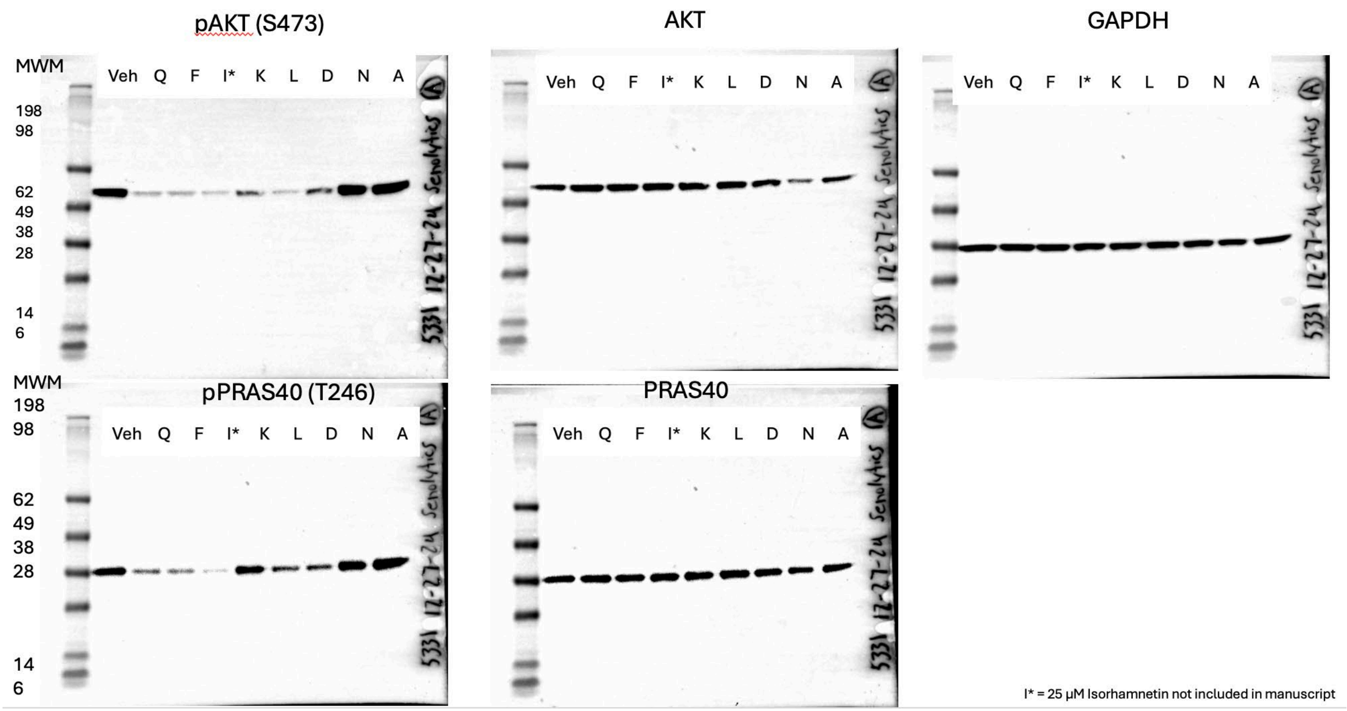

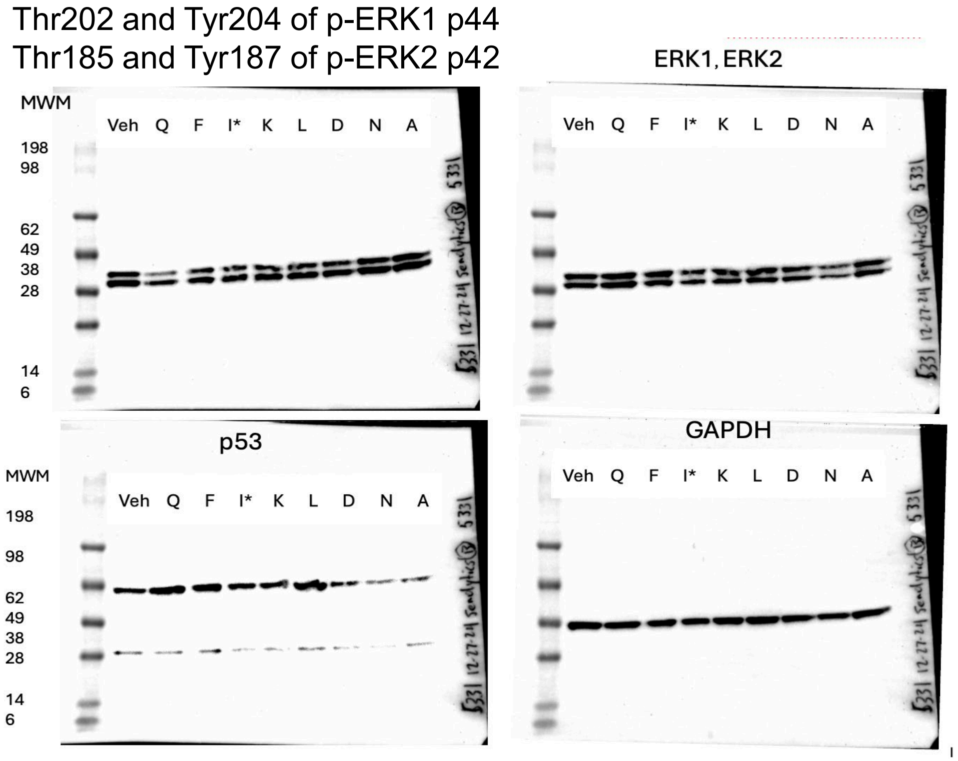

I* = 25 μM Isorhamnetin not included in manuscript

Figure 5. The differential effects of various flavonoid and non-flavonoid senotherapeutics on cell signaling pathways.

Participant #4

Supplemental data S 1

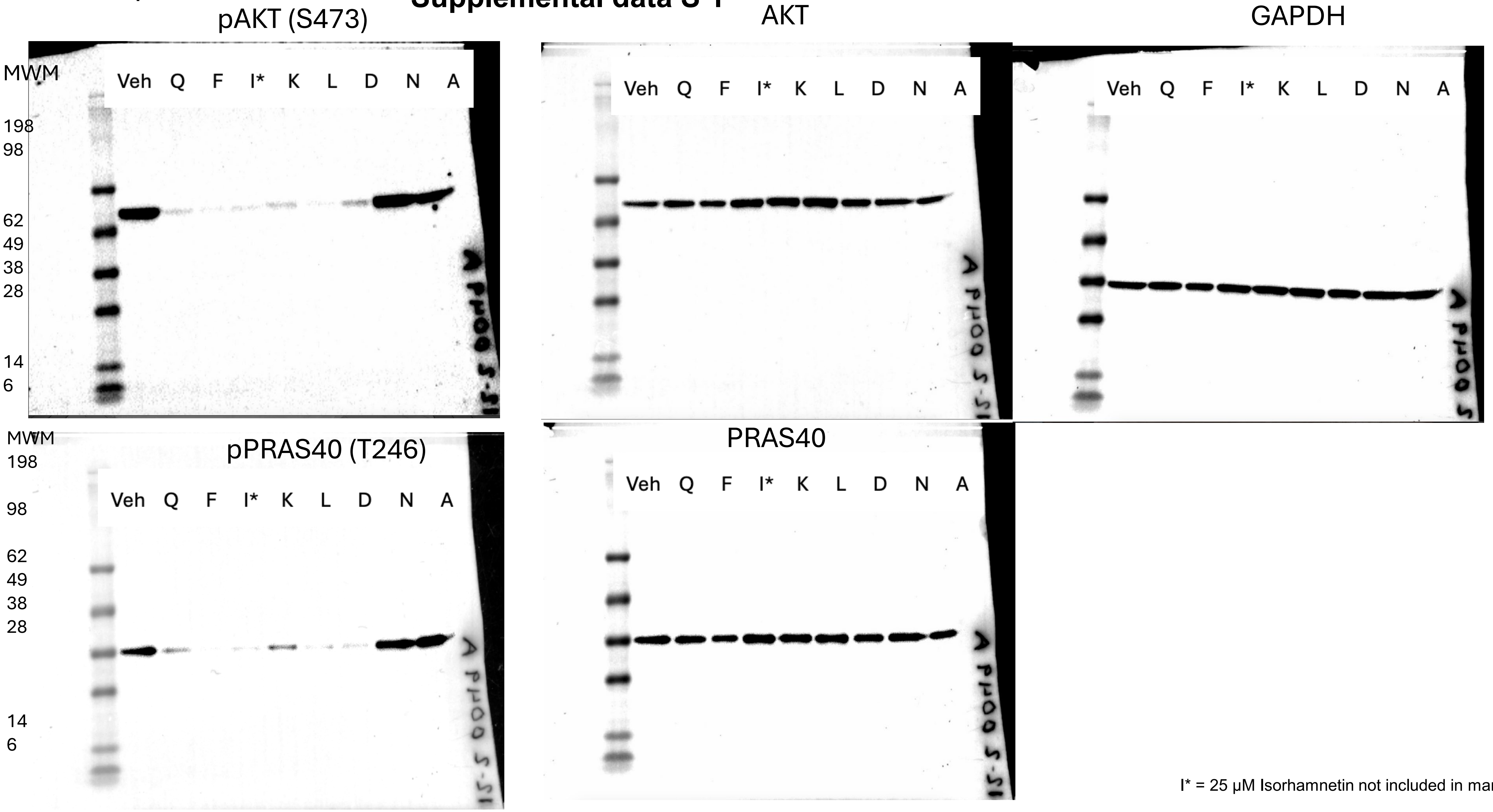

I* = 25 µM Isorhamnetin not included in manuscript

Figure 5. The differential effects of various flavonoid and non-flavonoid senotherapeutics on cell signaling pathways.

Supplemental data S 1

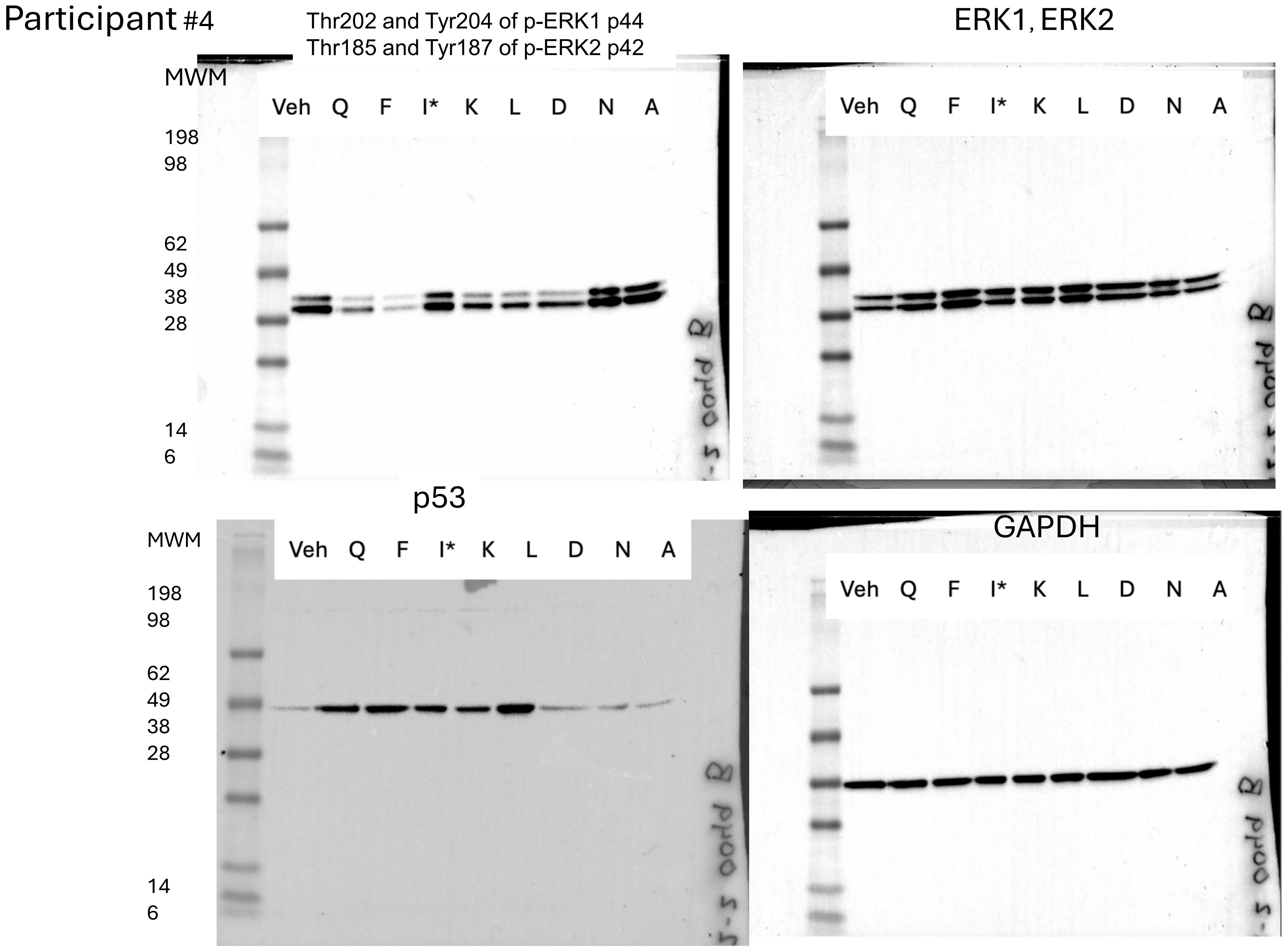

I* = 25 µM Isorhamnetin not included in manuscript

Supplemental data S 1

Figure 5. The differential effects of various flavonoid and non-flavonoid senotherapeutics on cell signaling pathways.

Participant #4

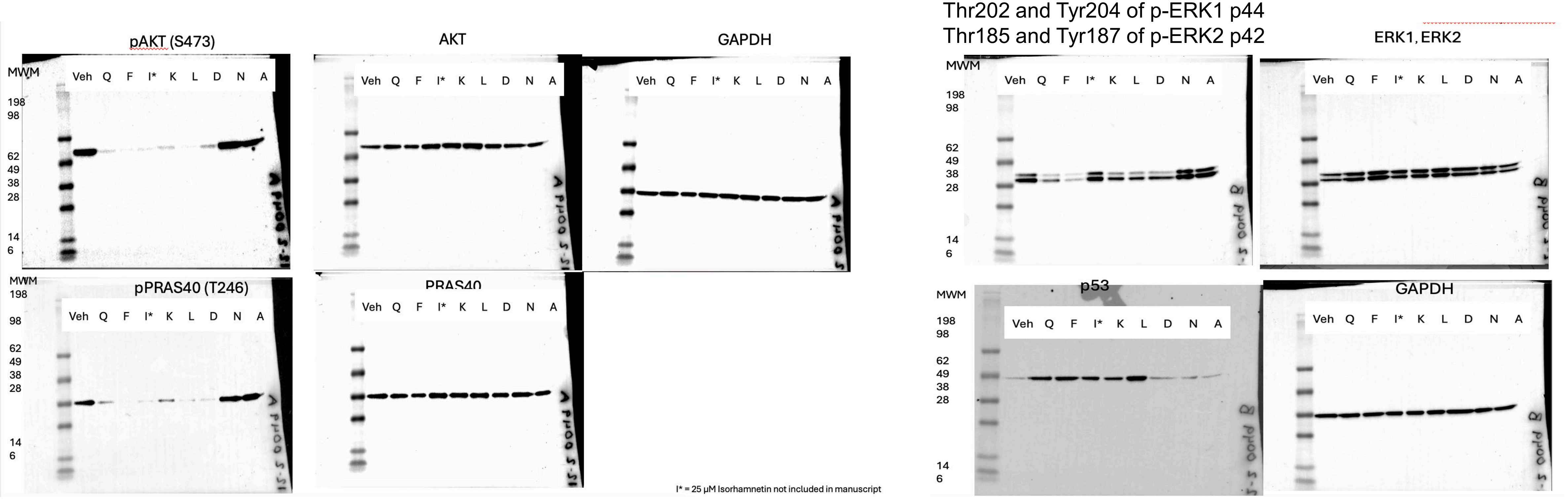

I* = 25 µM Isorhamnetin not included in manuscript

Figure 5. The differential effects of various flavonoid and non-flavonoid senotherapeutics on cell signaling pathways.

Supplemental data S 1

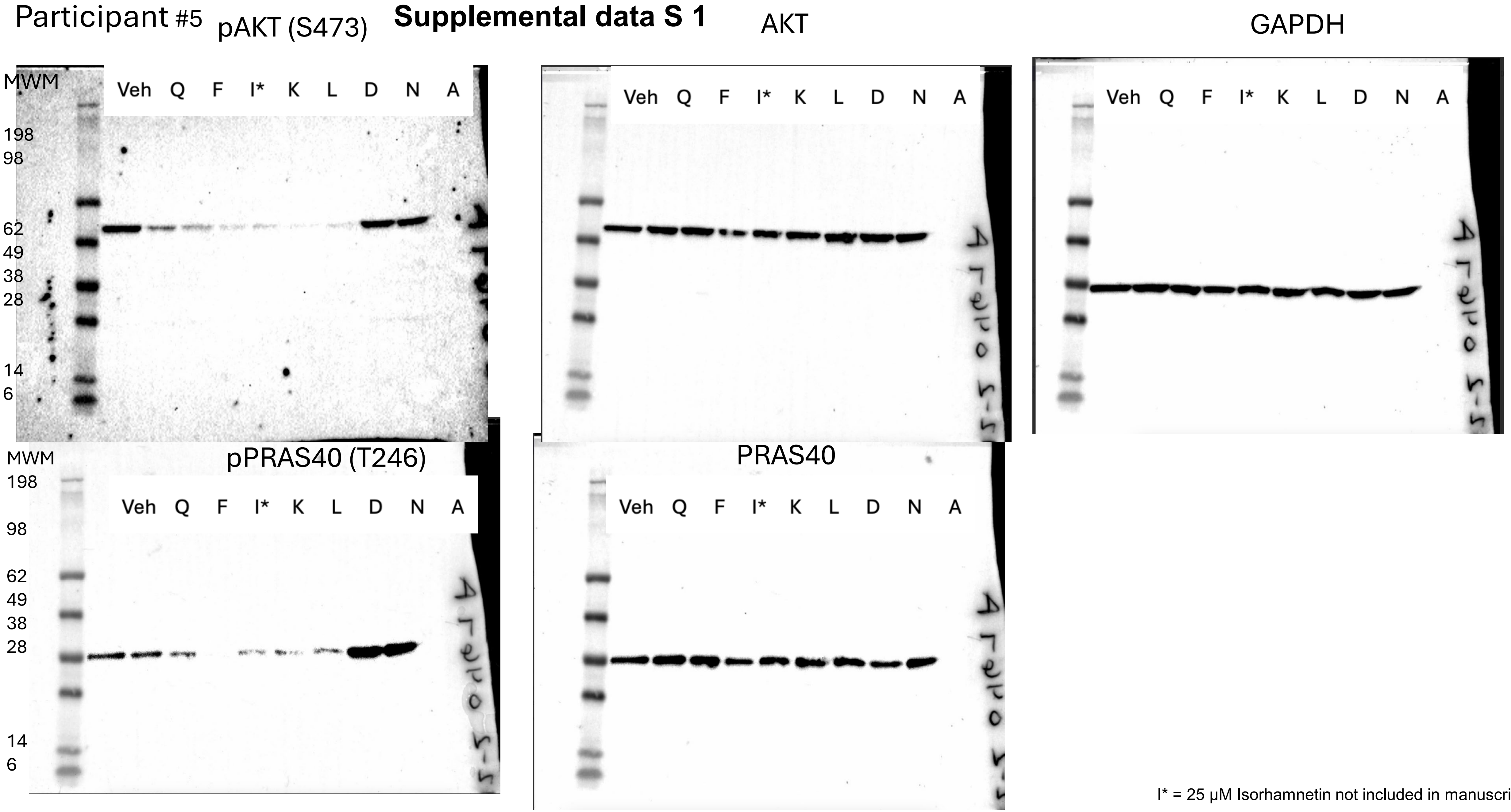

I* = 25 µM Isorhamnetin not included in manuscript

Figure 5. The differential effects of various flavonoid and non-flavonoid senotherapeutics on cell signaling pathways.

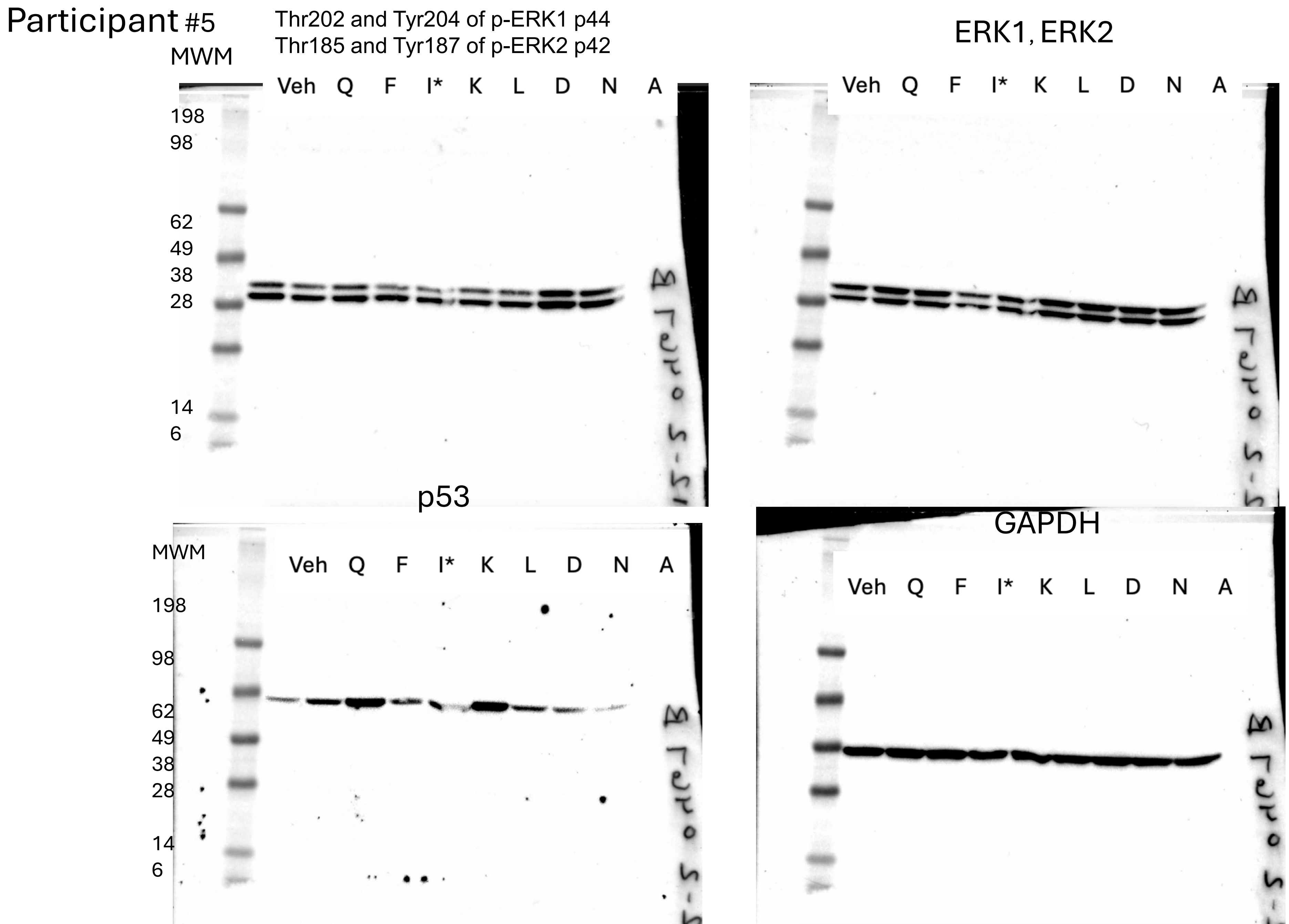

Supplemental data S 1

I* = 25 μM Isorhamnetin not included in manuscript

Supplemental data S 1

Figure 5. The differential effects of various flavonoid and non-flavonoid senotherapeutics on cell signaling pathways.
Participant #5

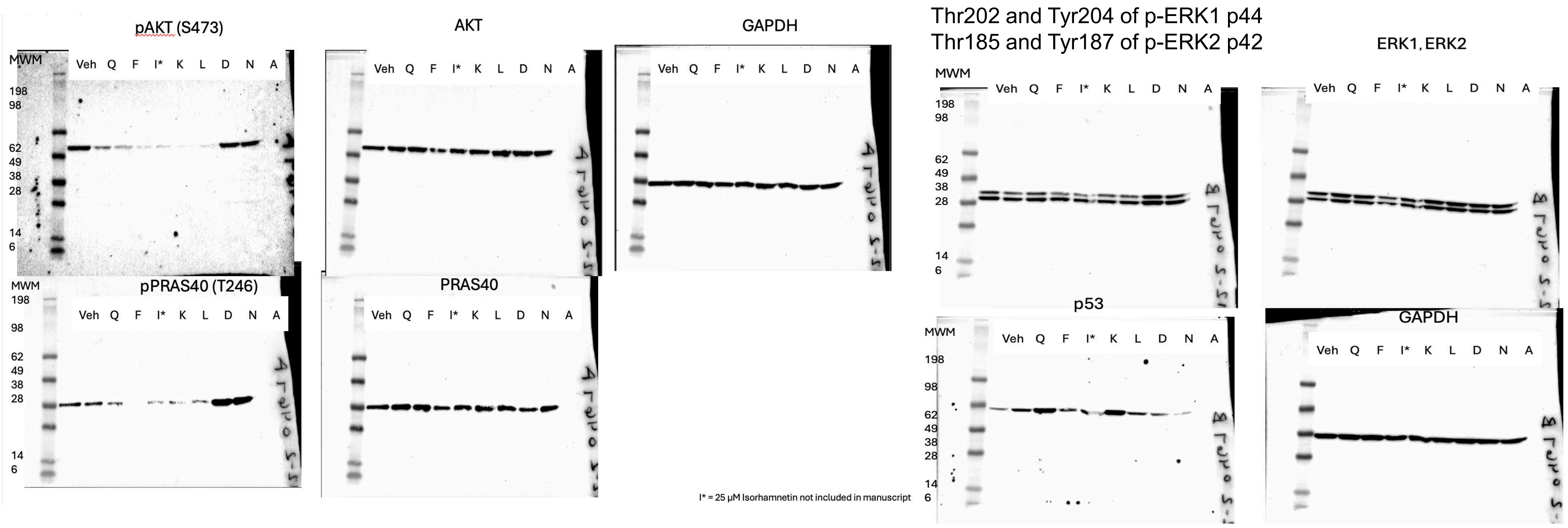

I* = 25 μM Isorhamnetin not included in manuscript

Figure 5. The differential effects of various flavonoid and non-flavonoid senotherapeutics on cell signaling pathways.

Supplemental data S 1

Participant #6

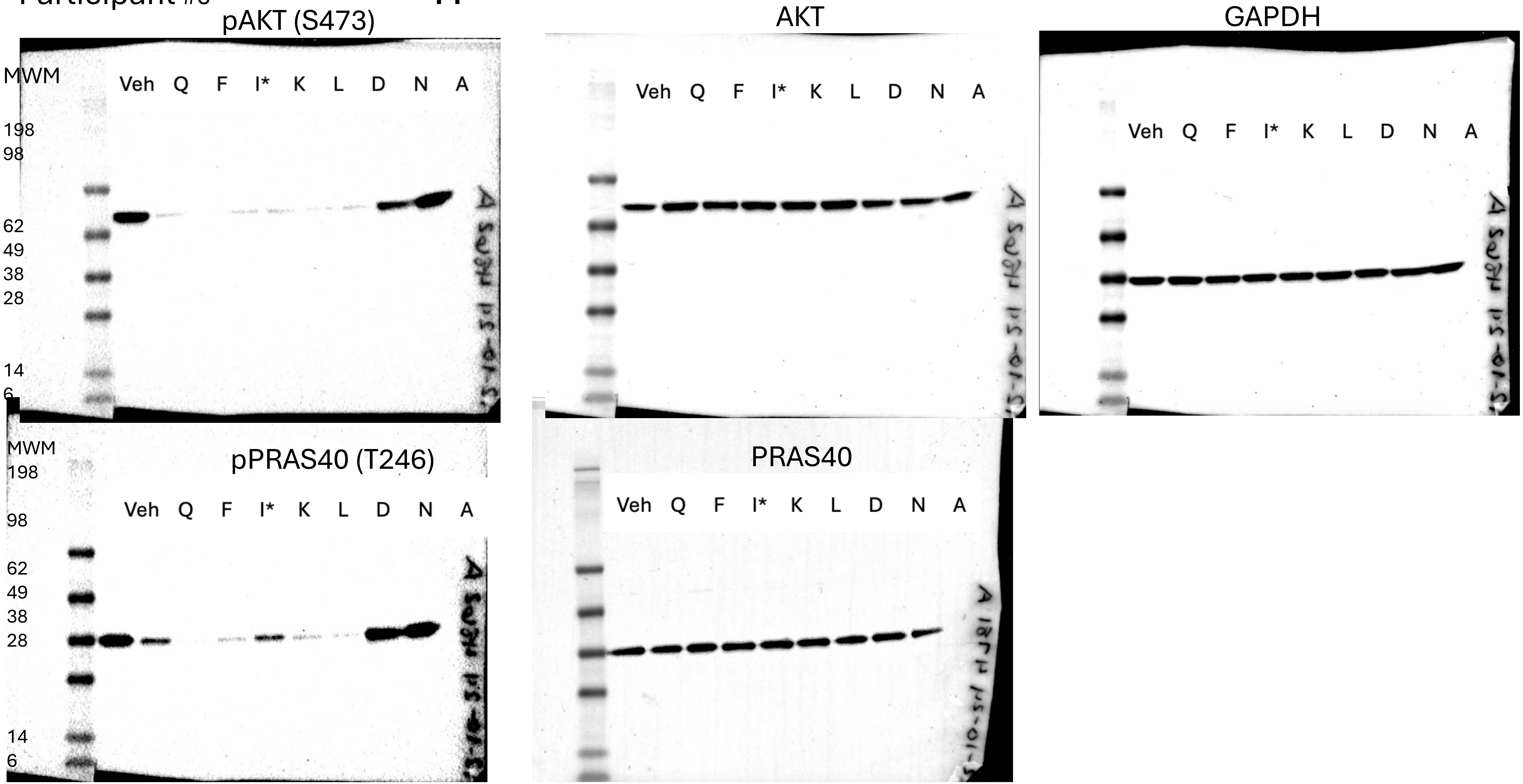

I* = 25 μM Isorhamnetin not included in manuscript

Figure 5. The differential effects of various flavonoid and non-flavonoid senotherapeutics on cell signaling pathways.

Participant #6

Supplemental data S 1

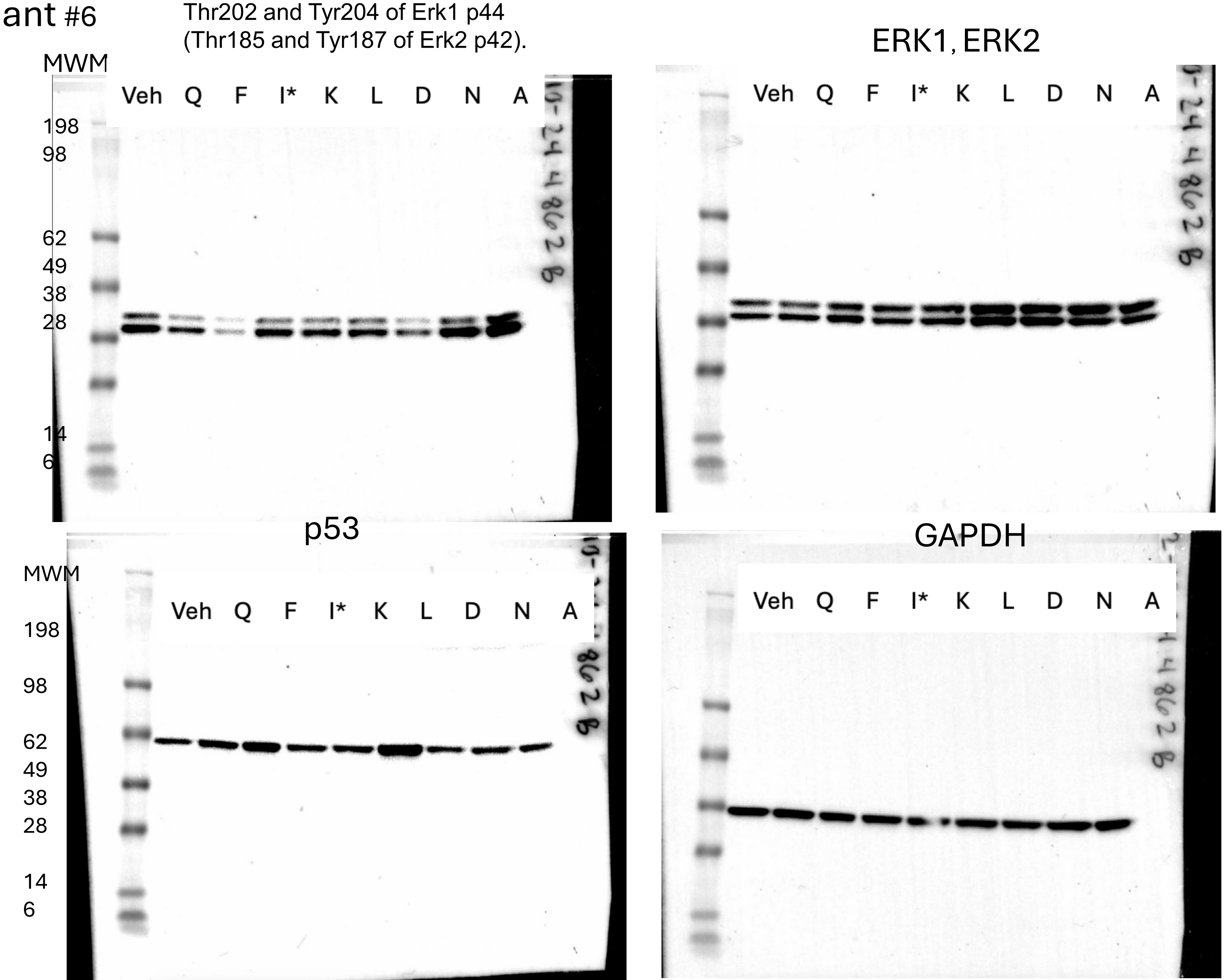

I* = 25 μM Isorhamnetin not included in manuscript

Supplemental data S 1

Figure 5. The differential effects of various flavonoid and non-flavonoid senotherapeutics on cell signaling pathways.

Participant #6

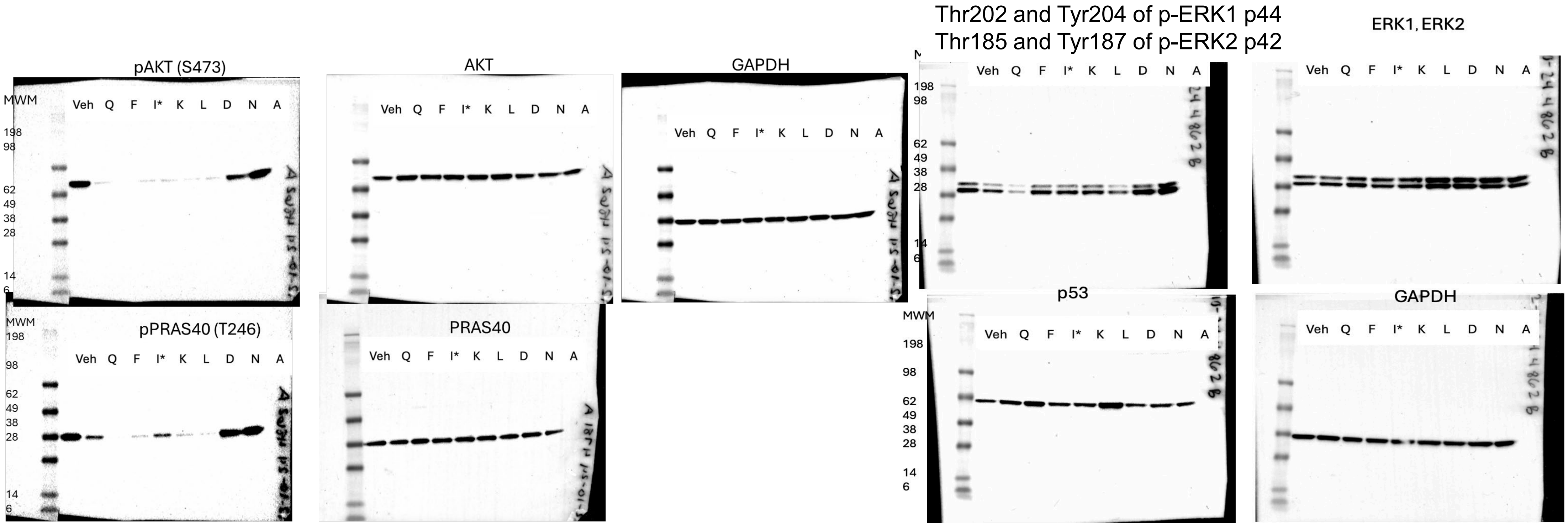

I* = 25 μM Isorhamnetin not included in manuscript

Figure 5. The differential effects of various flavonoid and non-flavonoid senotherapeutics on cell signaling pathways.

Supplemental data S 1

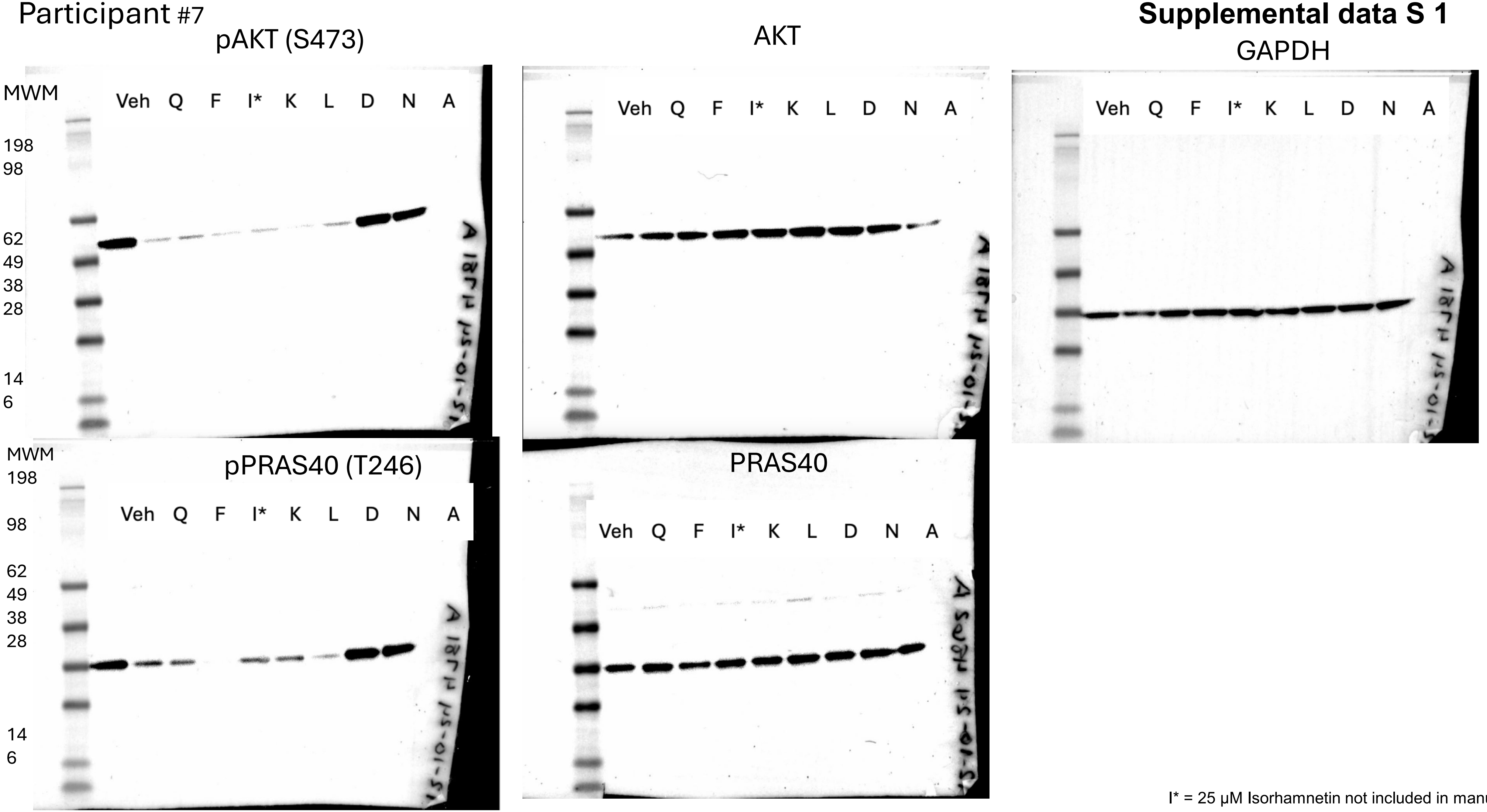

I* = 25 μM Isorhamnetin not included in manuscript

Figure 5. The differential effects of various flavonoid and non-flavonoid senotherapeutics on cell signaling pathways.

Supplemental data S 1

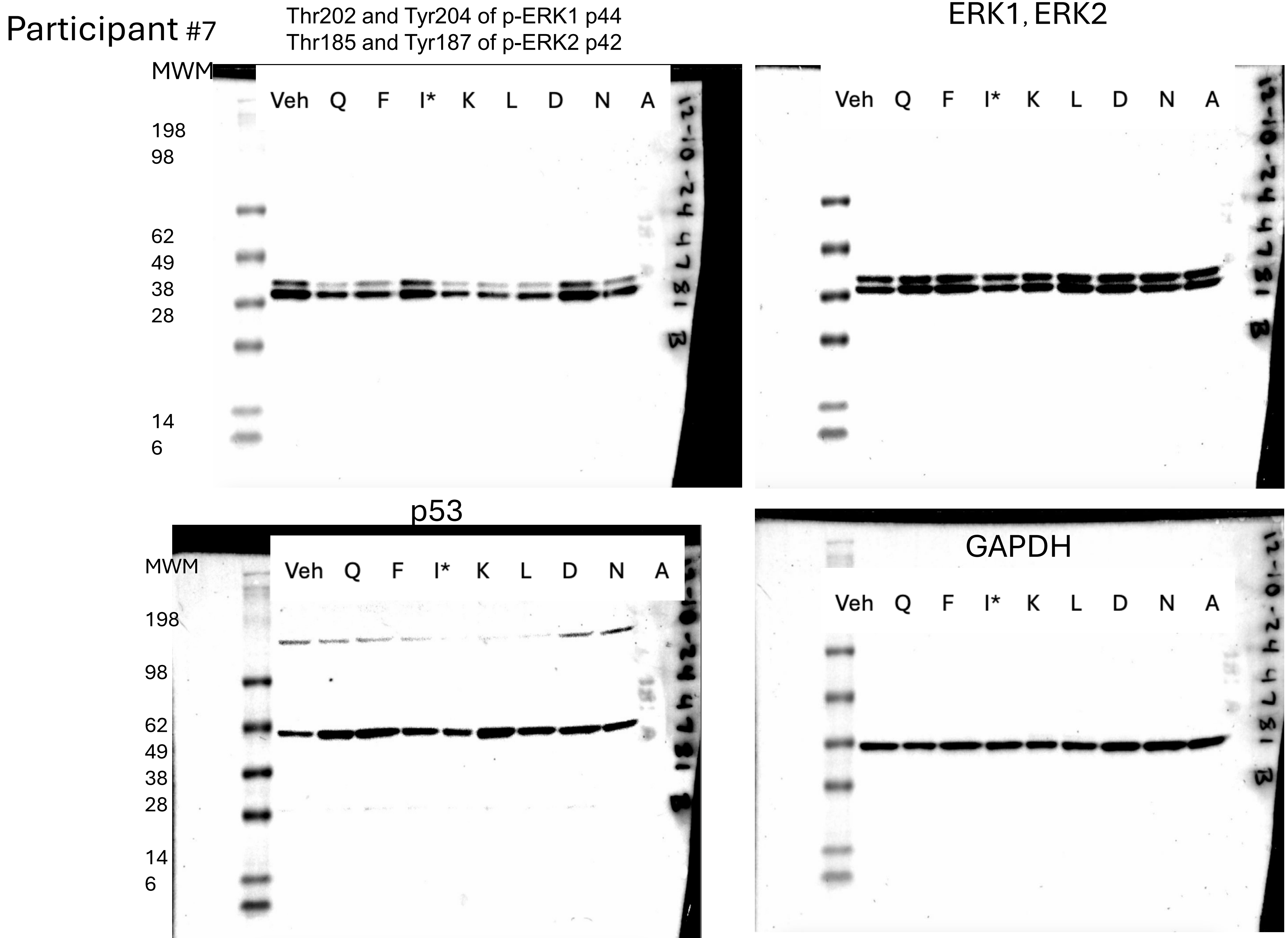

I* = 25 μM Isorhamnetin not included in manuscript

Supplemental data S 1

Figure 5. The differential effects of various flavonoid and non-flavonoid senotherapeutics on cell signaling pathways.

Participant #7

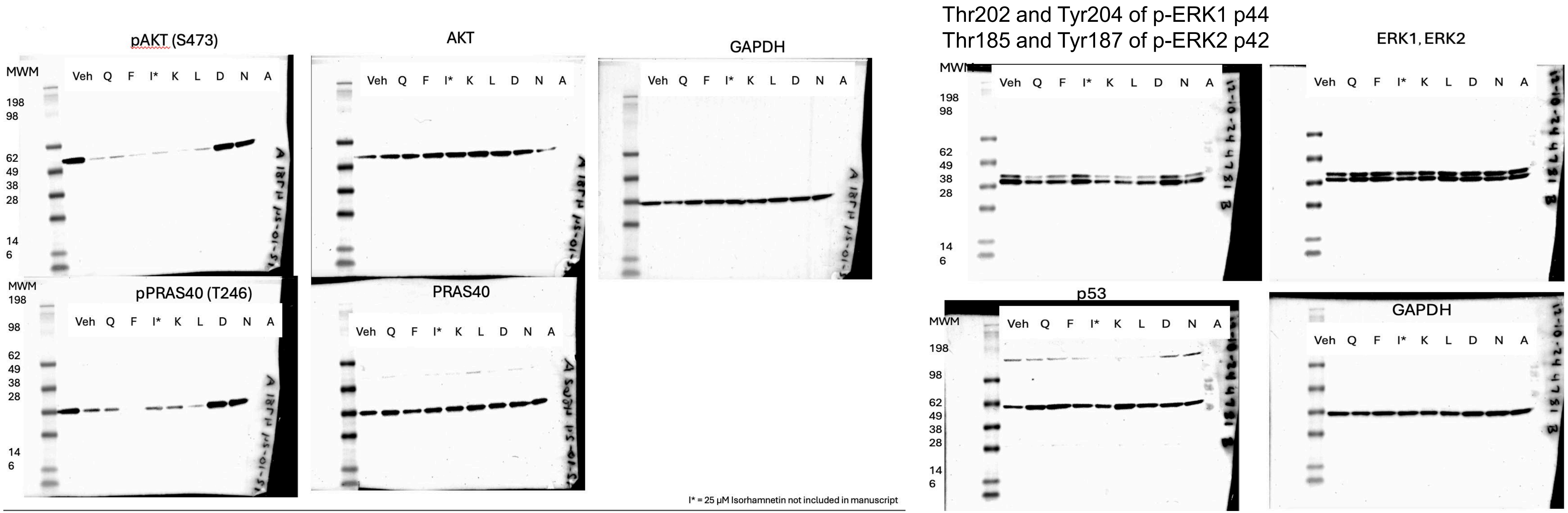

I* = 25 μM Isorhamnetin not included in manuscript

Figure 5. The differential effects of various flavonoid and non-flavonoid senotherapeutics on cell signaling pathways.

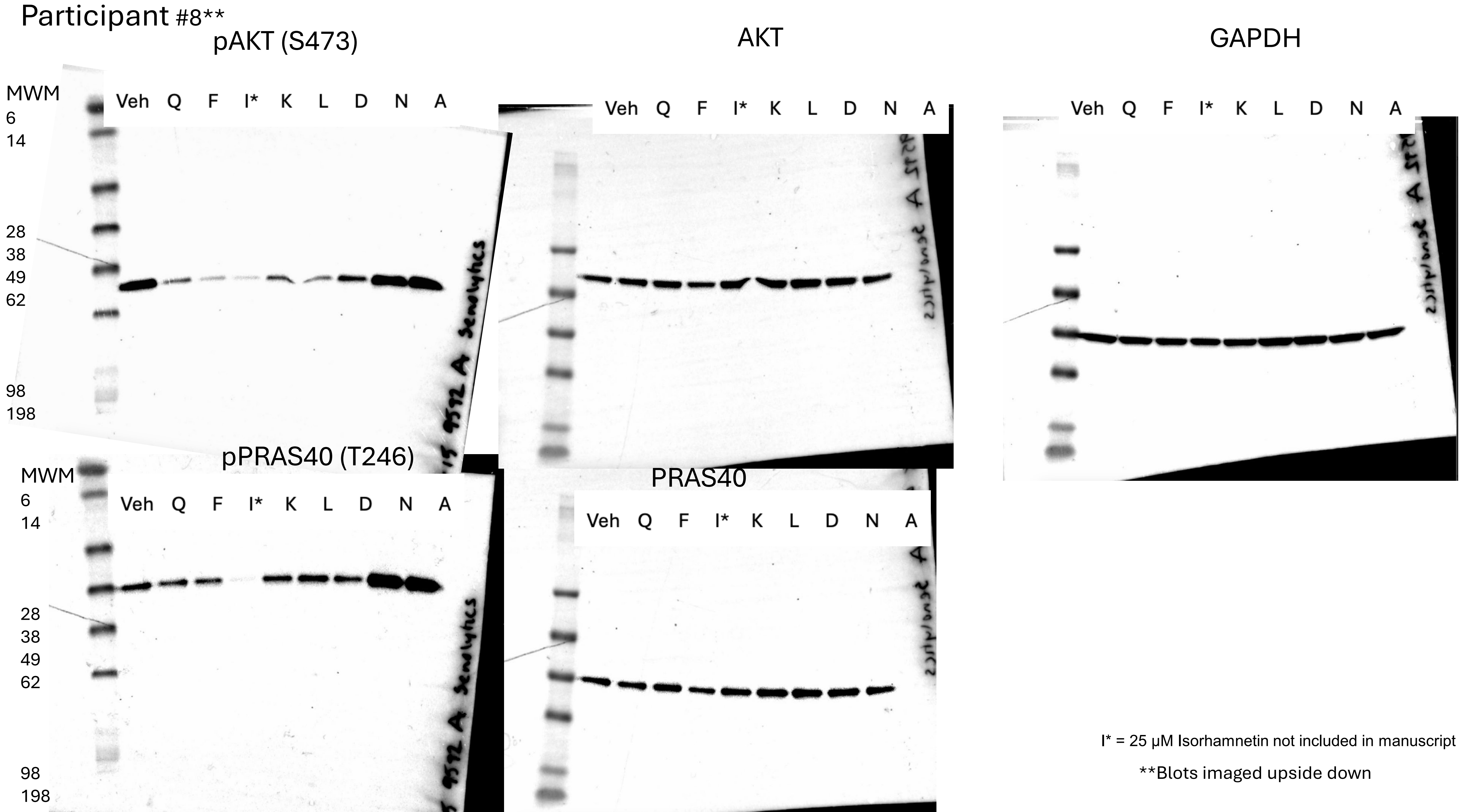

Figure 5. The differential effects of various flavonoid and non-flavonoid senotherapeutics on cell signaling pathways.

Participant #8**

Supplemental data S 1

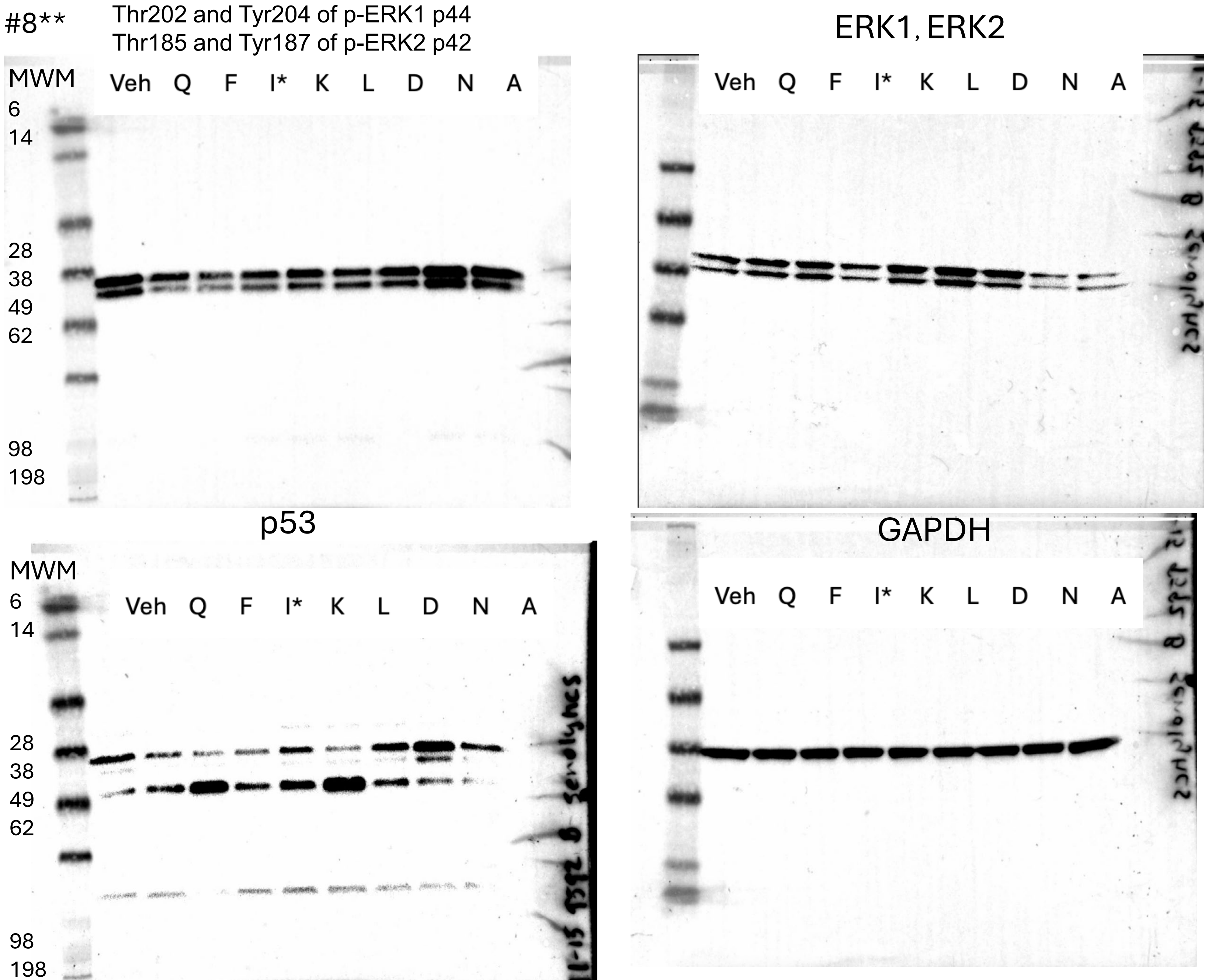

I* = 25 μM Isorhamnetin not included in manuscript

**Blots imaged upside down

Supplemental data S 1

Figure 5. The differential effects of various flavonoid and non-flavonoid senotherapeutics on cell signaling pathways.

Participant #8**

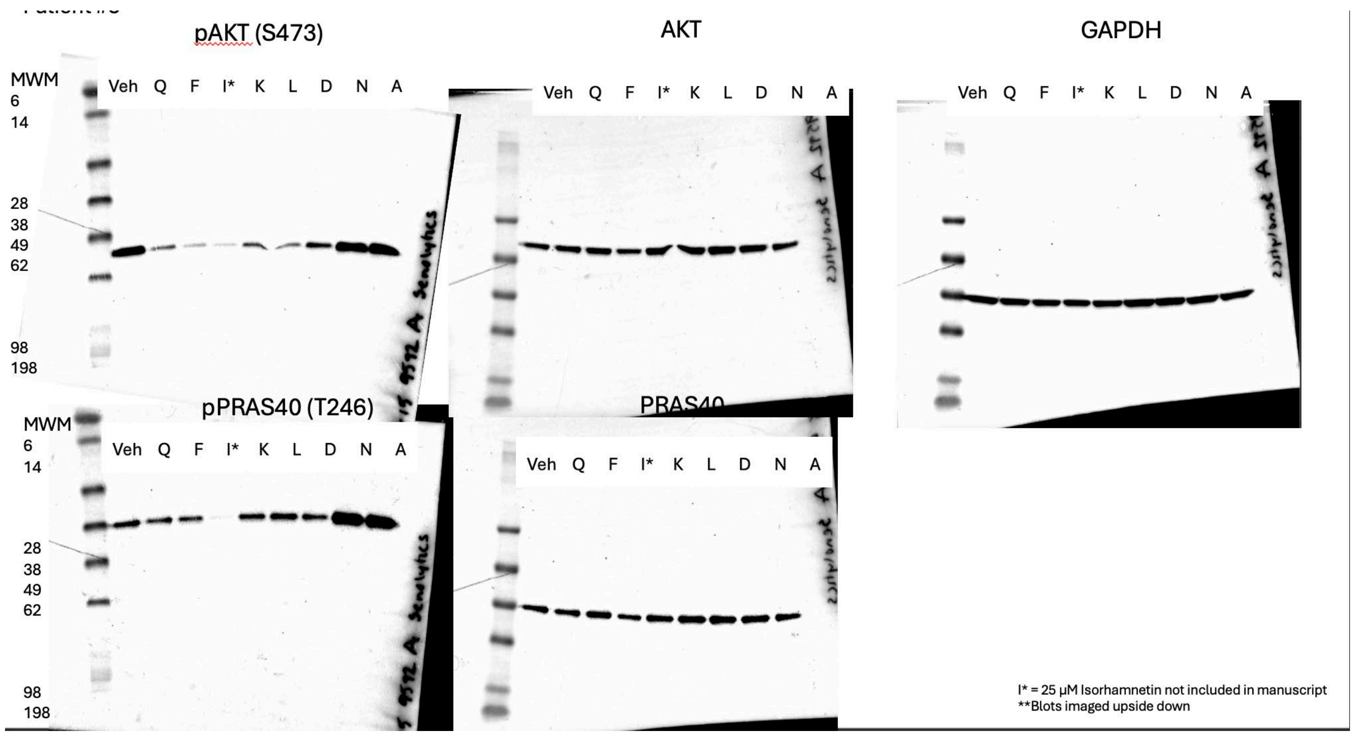

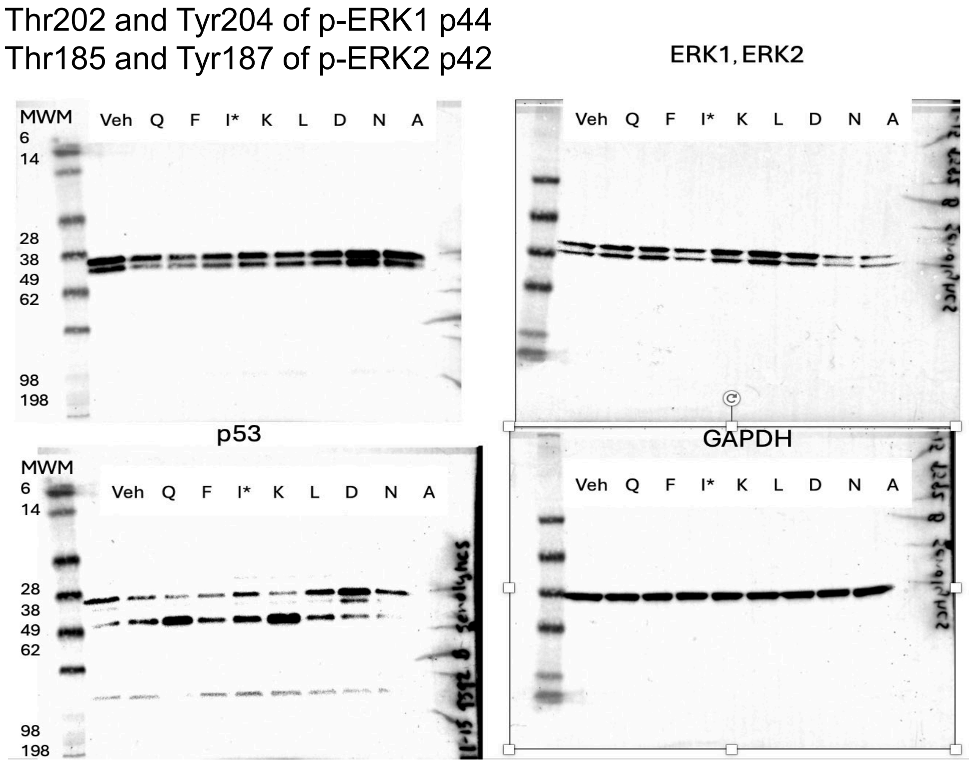

I* = 25 μM Isorhamnetin not included in manuscript

**Blots imaged upside down

All A blots for Figure 5

Supplemental Data S2:

Band densities and calculations for western blots shown in Figure 5.

See XLS spreadsheet for data (Tab: Supp2data western densities – left side for A blots)

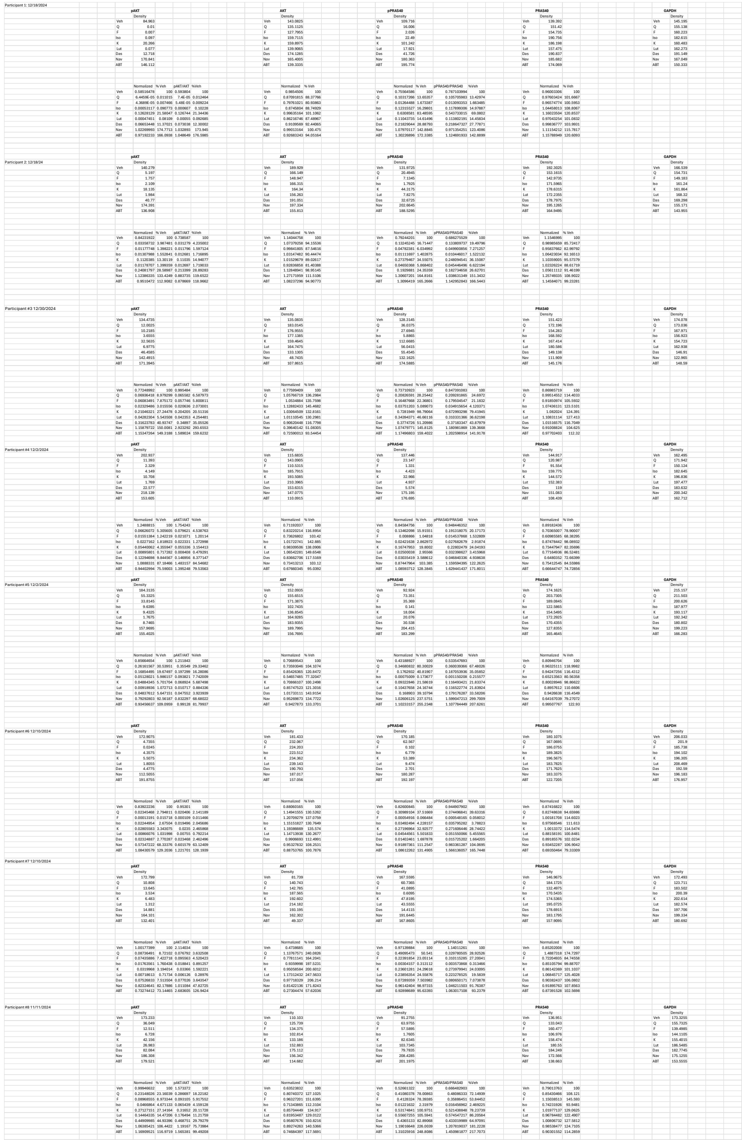

All B blots for Figure 5

Supplemental Data S2:

Band densities and calculations for western blots shown in Figure 5.

See XLS spreadsheet for data (Tab: Supp2data western densities – right side for B blots)

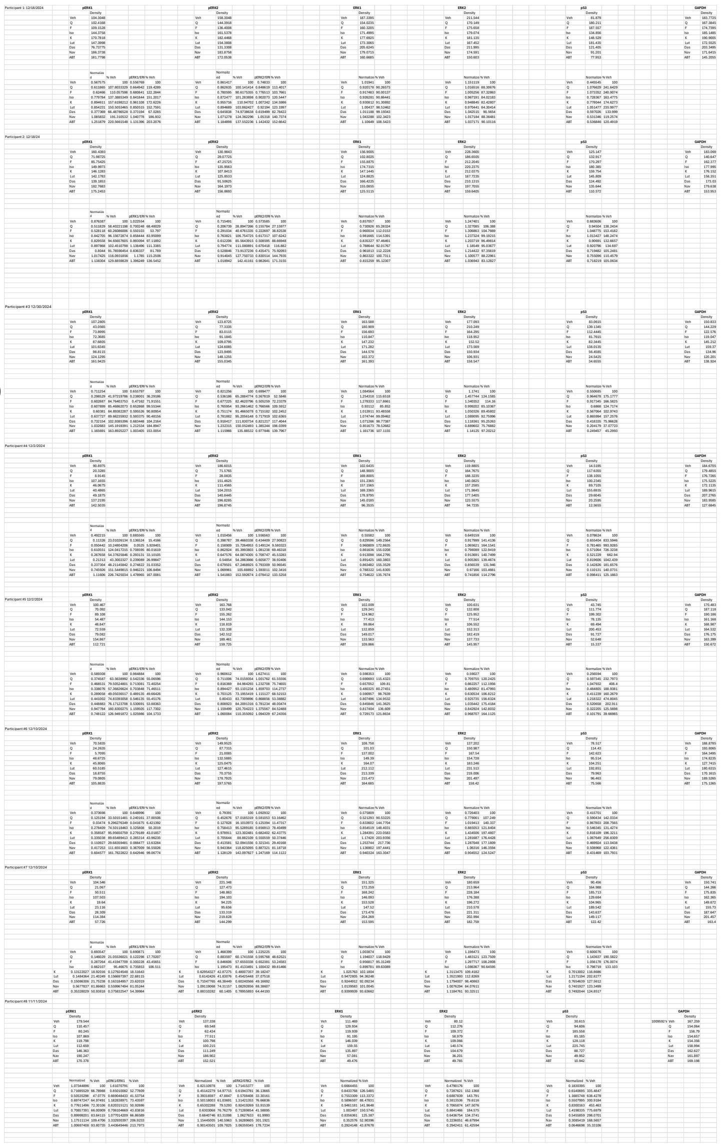

Supplemental Data S3: Western blot and band densities for eSCs obtained from endometriosis participants following treatment with vehicle vs. senotherapeutics.

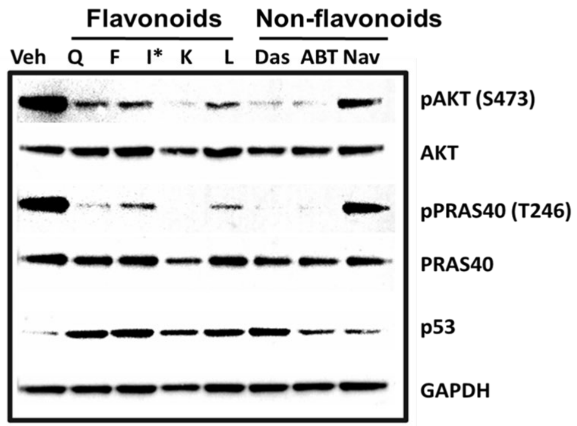

Patient Dx-ENDO1 2/27/25

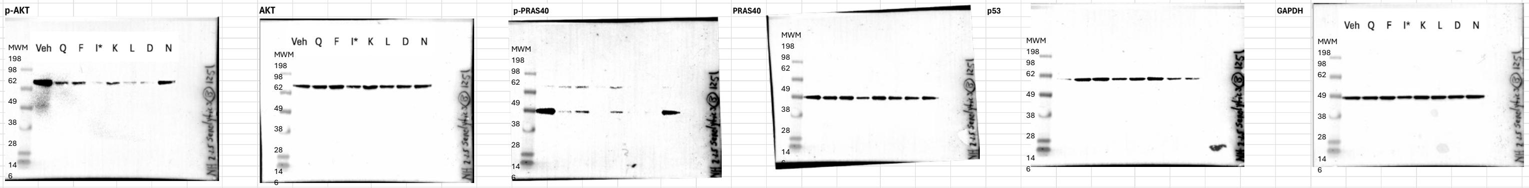

pAKT

	Density
Veh	161.9145
Q	8.3045
F	34.7195
Iso	0.1295
K	22.6155
Lut	1.5185
Das	1.7555
Nav	101.9525

	Normalized	% Veh	pPRAS/PRAS
Veh	1.06188413	100	1.085949
Q	0.05359661	5.047312	0.062344
F	0.21285355	20.04489	0.238403
Iso	0.00101773	0.095842	0.001791
K	0.14081749	13.2611	0.155533
Lut	0.00937169	0.882553	0.013516
Das	0.01115726	1.050704	0.01655
Nav	0.58216157	54.82345	0.895754

AKT

	Density
Veh	182.456
Q	50.134
F	32.384
Iso	0.1
K	21.509
Lut	0.1
Das	0.1
Nav	63.728

	Normalized	% Veh	pAKT/AKT
Veh	1.19660149	100	1.348519
Q	0.32356102	27.04	0.326132
F	0.19853538	16.5916	0.1884
Iso	0.00078589	0.065677	0.00093
K	0.13392777	11.19234	0.132433
Lut	0.00061717	0.051577	0.000826
Das	0.00063556	0.053114	0.00074
Nav	0.36389488	30.4107	0.422465

pPRAS40

	Density
Veh	149.0995
Q	133.2045
F	145.6335
Iso	72.3045
K	145.4065
Lut	112.3485
Das	106.0705
Nav	113.8175

	Normalized	% Veh
Veh	0.9778395	100
Q	0.8596917	87.91746
F	0.89282988	91.30638
Iso	0.56823281	58.11105
K	0.90538694	92.59055
Lut	0.69337872	70.90926
Das	0.67414191	68.94198
Nav	0.64991221	66.4641

PRAS40

	Density
Veh	135.301
Q	153.723
F	171.89
Iso	107.495
K	162.414
Lut	121.023
Das	135.09
Nav	150.848

	Normalized	% Veh
Veh	0.88734477	100
Q	0.99211653	111.8073
F	1.05379963	118.7588
Iso	0.84479093	95.20436
K	1.0112857	113.9676
Lut	0.74691493	84.17415
Das	0.85857832	96.75814
Nav	0.86136101	97.07174

p53

	Density
Veh	12.234
Q	151.375
F	166.851
Iso	94.663
K	133.016
Lut	152.449
Das	65.927
Nav	47.63

	Normalized	% Veh
Veh	0.08023426	100
Q	0.97696272	1217.638
F	1.02290722	1274.901
Iso	0.74394571	927.217
K	0.82823635	1032.273
Lut	0.94086607	1172.649
Das	0.41900579	522.228
Nav	0.27197328	338.974

GAPDH

	Density
Veh	152.4785
Q	154.9445
F	163.1145
Iso	127.2445
K	160.6015
Lut	162.0305
Das	157.3415
Nav	175.1275
ABT	139.5125

Note: I* = isohamnetin, not included in manuscript

Supplemental Data S5: Western blot and band densities for senescence studies with eSCs, including NanoJagg-positive (NJ+, senescent cells) and NanoJagg-negative (NJ-, non-senescent cells) and eSCs trated with vehicle (Veh) or quercetin (Q).

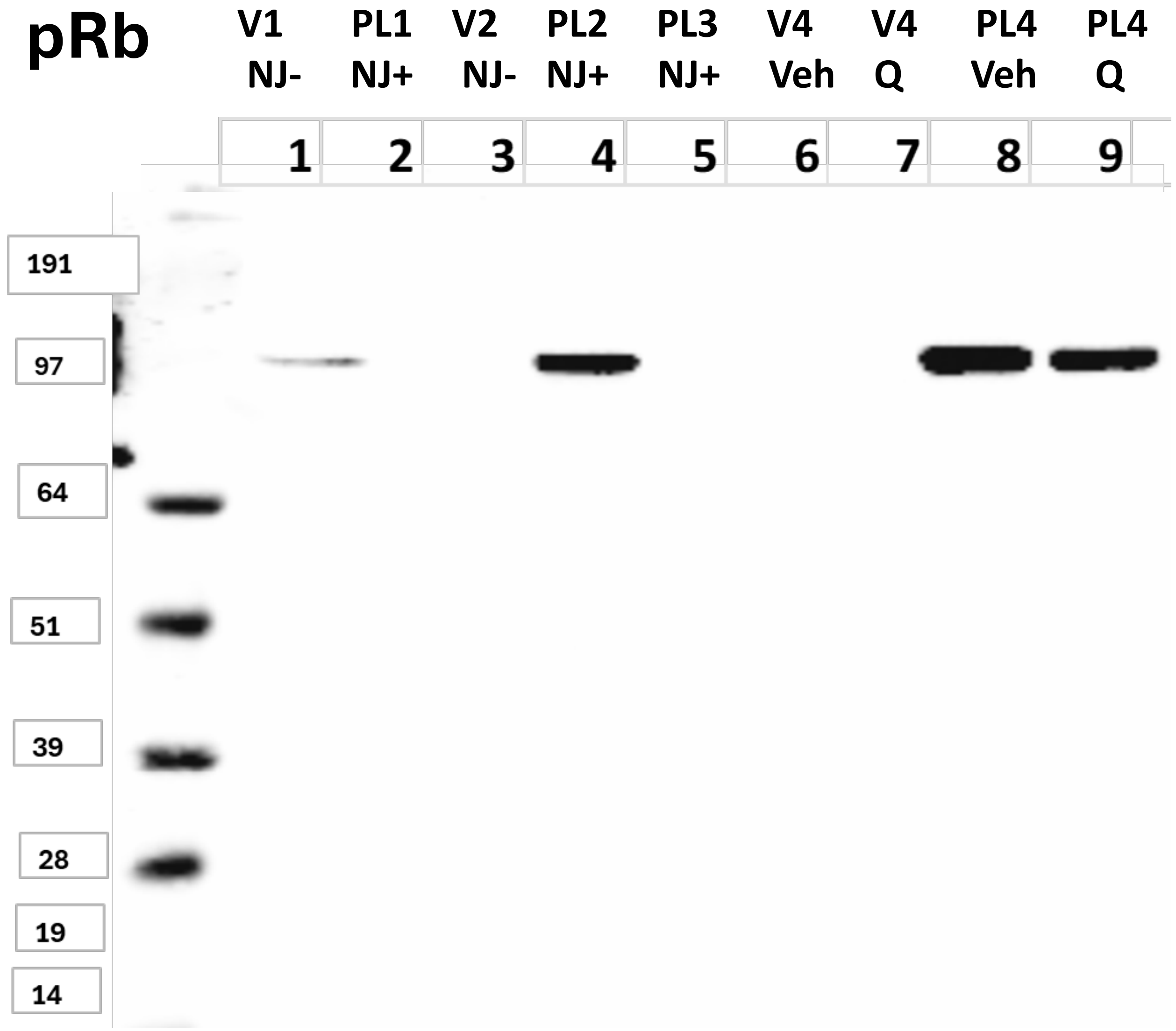

	p-Rb	GAPDH	p-Rb/GAPDH
1	17.93	165.36	0.11
2	1.02	168.42	0.01
3	129.01	160.61	0.80
4	1.00	166.25	0.01
5	1.00	130.62	0.01
6	185.35	162.43	1.14
7	149.25	168.11	0.89
8	1.02	179.65	0.01
9	1.00	171.65	0.01

Supplemental Data S5: Western blot and band densities for senescence studies with eSCs, including NanoJagg-positive (NJ+, senescent cells) and NanoJagg-negative (NJ-, non-senescent cells) and eSCs trated with vehicle (Veh) or quercetin (Q).

	p16	GAPDH	p16/GAPDH
1	68.20	166.26	0.410
2	111.06	169.31	0.656
3	13.35	161.50	0.083
4	117.89	167.15	0.705
5	127.92	131.52	0.973
6	211.27	163.32	1.294
7	158.31	169.01	0.937
8	21.55	180.55	0.119
9	1.67	172.55	0.010

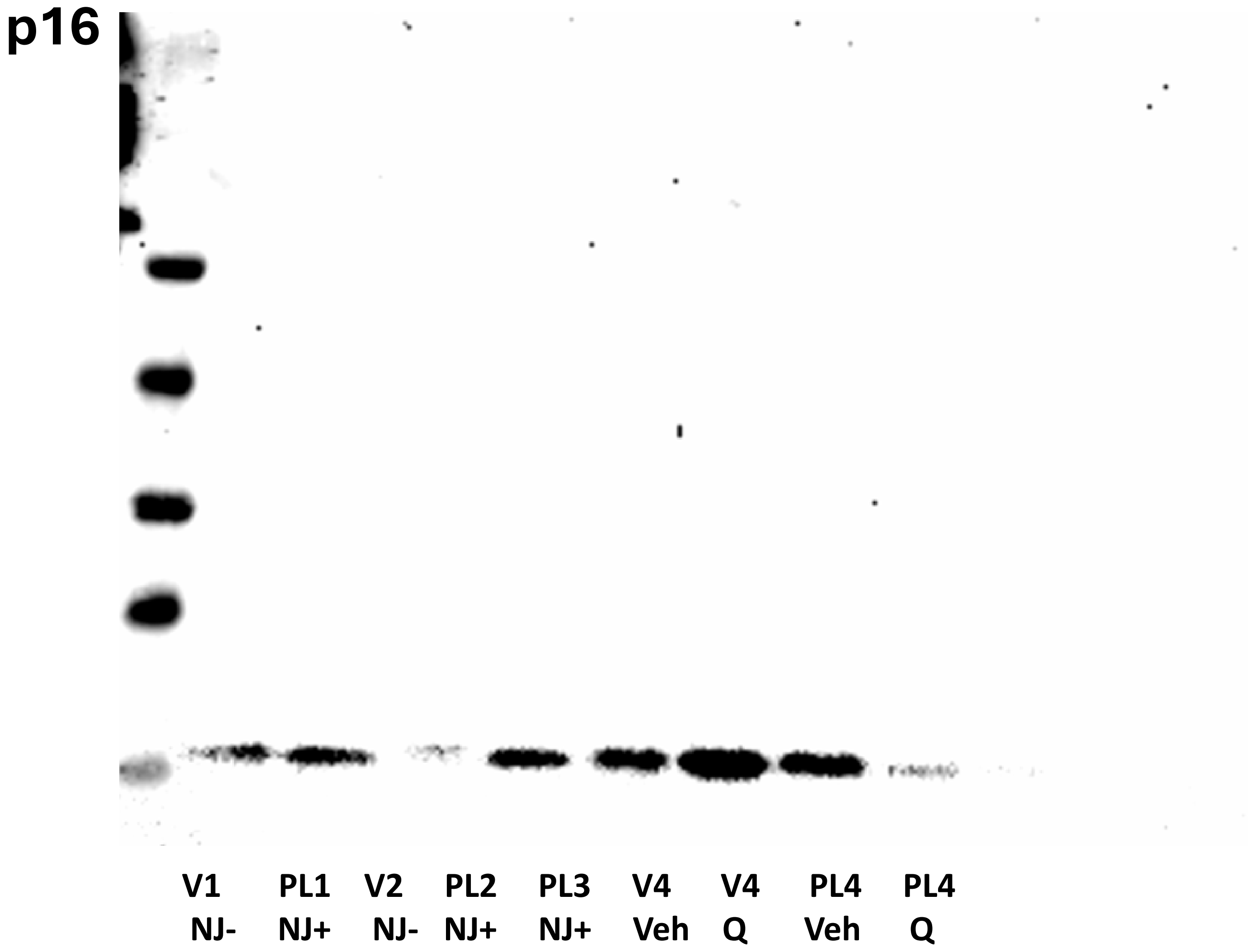

Supplemental Data S5: Western blot and band densities for senescence studies with eSCs, including NanoJagg-positive (NJ+, senescent cells) and NanoJagg-negative (NJ-, non-senescent cells) and eSCs trated with vehicle (Veh) or quercetin (Q).

	GAPDH	GAPDH	GAPDH/GAPDH
1	166.26	166.26	1
2	169.31	169.31	1
3	161.50	161.50	1
4	167.15	167.15	1
5	131.52	131.52	1
6	163.32	163.32	1
7	169.01	169.01	1
8	180.55	180.55	1
9	172.55	172.55	1

GAPDH

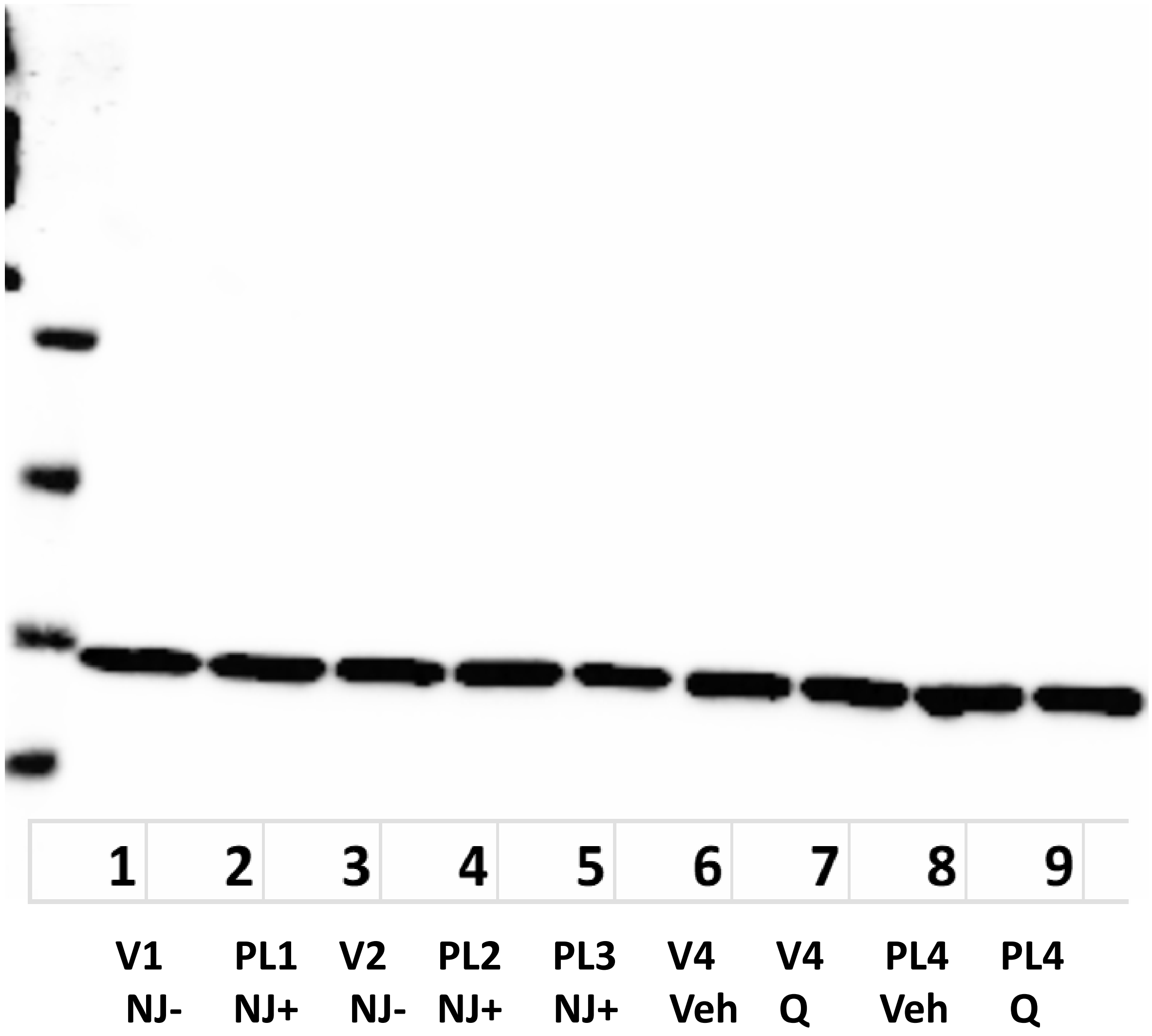

Supplement: Supplementary file 1 [file biomolecules-15-00873-s001.zip › SUPP-Blot-Data_Delenko-6-11-25.pdf]
